# Ameliorating the hallmarks of cellular senescence in skeletal muscle myogenic progenitors in vitro and in vivo

**DOI:** 10.1126/sciadv.abe5671

**Published:** 2021-09-03

**Authors:** Aref Shahini, Nika Rajabian, Debanik Choudhury, Shahryar Shahini, Kalyan Vydiam, Thy Nguyen, Joseph Kulczyk, Tyler Santarelli, Izuagie Ikhapoh, Yali Zhang, Jianmin Wang, Song Liu, Aimee Stablewski, Ramkumar Thiyagarajan, Kenneth Seldeen, Bruce R. Troen, Jennifer Peirick, Pedro Lei, Stelios T. Andreadis

**Affiliations:** 1 Bioengineering Laboratory, Department of Chemical and Biological Engineering, University at Buffalo, The State University of New York, Buffalo, NY 14260, USA.; 2 Department of Biomedical Engineering, University at Buffalo, The State University of New York, Buffalo, NY 14260, USA.; 3 Department of Biostatistics and Bioinformatics, Roswell Park Cancer Institute, Buffalo, NY 14260, USA.; 4 Gene Targeting and Transgenic Shared Resource, Roswell Park Comprehensive Cancer Center.; 5 Department of Medicine, Jacobs School of Medicine and Biomedical Sciences, University at Buffalo, The State University of New York, Buffalo, NY 14260, USA.; 6 Research Service, VA Western New York Healthcare System, Buffalo, NY 14260, USA.; 7 Laboratory Animal Facilities, University at Buffalo, The State University of New York, Buffalo, NY 14260, USA.; 8 Center of Excellence in Bioinformatics and Life Sciences, University at Buffalo, The State University of New York, Buffalo, NY 14260, USA.; 9 Center for Cell Gene and Tissue Engineering (CGTE), University at Buffalo, The State University of New York, Buffalo, NY 14260, USA.

## Abstract

Senescence of myogenic progenitors impedes skeletal muscle regeneration. Here, we show that overexpression of the transcription factor NANOG in senescent myoblasts can overcome the effects of cellular senescence and confer a youthful phenotype to senescent cells. NANOG ameliorated primary hallmarks of cellular senescence including genomic instability, loss of proteostasis, and mitochondrial dysfunction. The rejuvenating effects of NANOG included restoration of DNA damage response via up-regulation of DNA repair proteins, recovery of heterochromatin marks via up-regulation of histones, and reactivation of autophagy and mitochondrial energetics via up-regulation of AMP-activated protein kinase (AMPK). Expression of NANOG in the skeletal muscle of a mouse model of premature aging restored the number of myogenic progenitors and induced formation of eMyHC^+^ myofibers. This work demonstrates the feasibility of reversing the effects of cellular senescence in vitro and in vivo, with no need for reprogramming to the pluripotent state.

## Teaser

The embryonic factor NANOG reverses aging hallmarks and rejuvenates senescent skeletal muscle progenitors.

## INTRODUCTION

Cellular senescence due to aging or chronic disease impedes stem cell function and regeneration (*1*). Skeletal muscle is a highly regenerative organ that comprises ~45% of body mass and enables skeletal movements while also regulating metabolism. Muscle regeneration relies on myogenic progenitors that, when activated, proliferate, differentiate, and contribute to the regeneration of damaged myofibers. However, both the number of myogenic progenitors and their regenerative capacity decline with aging and cellular senescence (*2*).

In general, genomic instability, telomere attrition, epigenetic alterations, and loss of proteostasis are recognized as primary aging hallmarks that result in deregulated nutrient sensing, mitochondrial dysfunction, and, ultimately, cellular senescence (*3*). Senescent cells stop proliferating because of genomic instability and telomere attrition, increase in size because of loss of proteostasis, and lose energy homeostasis because of mitochondrial dysfunction (*3*–*5*). To ameliorate cellular senescence, previous research has targeted intracellular and extracellular signaling pathways and partial reprogramming to induce a youthful phenotype in senescent cells (*1*, *3*–*5*).

Hutchinson-Gilford progeria syndrome or progeria is a disease leading to premature aging due to a mutation in the *Lamin A* (*LMNA*) gene, resulting in aberrant accumulation of a truncated form of LMNA protein, otherwise known as progerin. To elicit premature aging, progerin induces epigenetic changes (*6*), DNA damage (*7*), and oxidative stress (*8*), the same factors that induce cellular senescence over multiple population doublings (*9*–*11*). *Lmna* knock-in (LAKI) mice show symptoms of premature aging, including musculoskeletal and cardiovascular aberrations and a shorter life span (*12*). In addition, progerin levels increase in normal aging due to the sporadic use of the LMNA cryptic splice site in healthy individuals and contributes to cellular senescence (*13*). For these reasons, the progeroid mouse model has become a well-accepted animal model to study aging and evaluate anti-aging therapies (*12*, *14*, *15*).

Cellular rejuvenation occurs naturally in embryonic development when sperm and egg (each having a certain chronological age) fuse to each other to form an embryo of age zero (*16*). Similarly, reprogramming of somatic cells to pluripotency [induced pluripotent stem cells (iPSCs)] resets their biological clock as well (*17*). At this stage, a core network of transcription factors including NANOG, OCT4, and SOX2 maintains pluripotency in embryonic stem cells (ESCs) and iPSCs (*18*). In particular, the pluripotency factor NANOG is essential for maintaining the self-renewal of ESCs over many population doublings (*19*). Although overexpression of NANOG does not confer pluripotency to somatic cells, it has been shown to restore several cellular functions that are compromised by aging including proliferation and differentiation of senescent fibroblasts and mesenchymal stem cells (*20*–*22*). In vivo endogenous expression of this transcription factor in stratified epithelia of adult mice showed that systemic overexpression of NANOG induces hyperplasia without initiating tumors (*23*–*26*).

Recently, we discovered that expression of NANOG in C2C12 myoblasts restored their myogenic differentiation potential, as evidenced by expression of myogenic regulatory factors and the ability to form myotubes, which was impaired by replicative senescence (*27*). This result prompted us to investigate the anti-aging effects of NANOG on primary human myoblasts and in skeletal muscle tissue in vivo. Here, we show that overexpression of NANOG reversed the hallmarks of cellular senescence in muscle progenitors in vitro and restored the satellite cell abundance in the skeletal muscle of LAKI progeria mice.

## RESULTS

### Overexpression of NANOG in human myoblasts

**Fig. 1.**
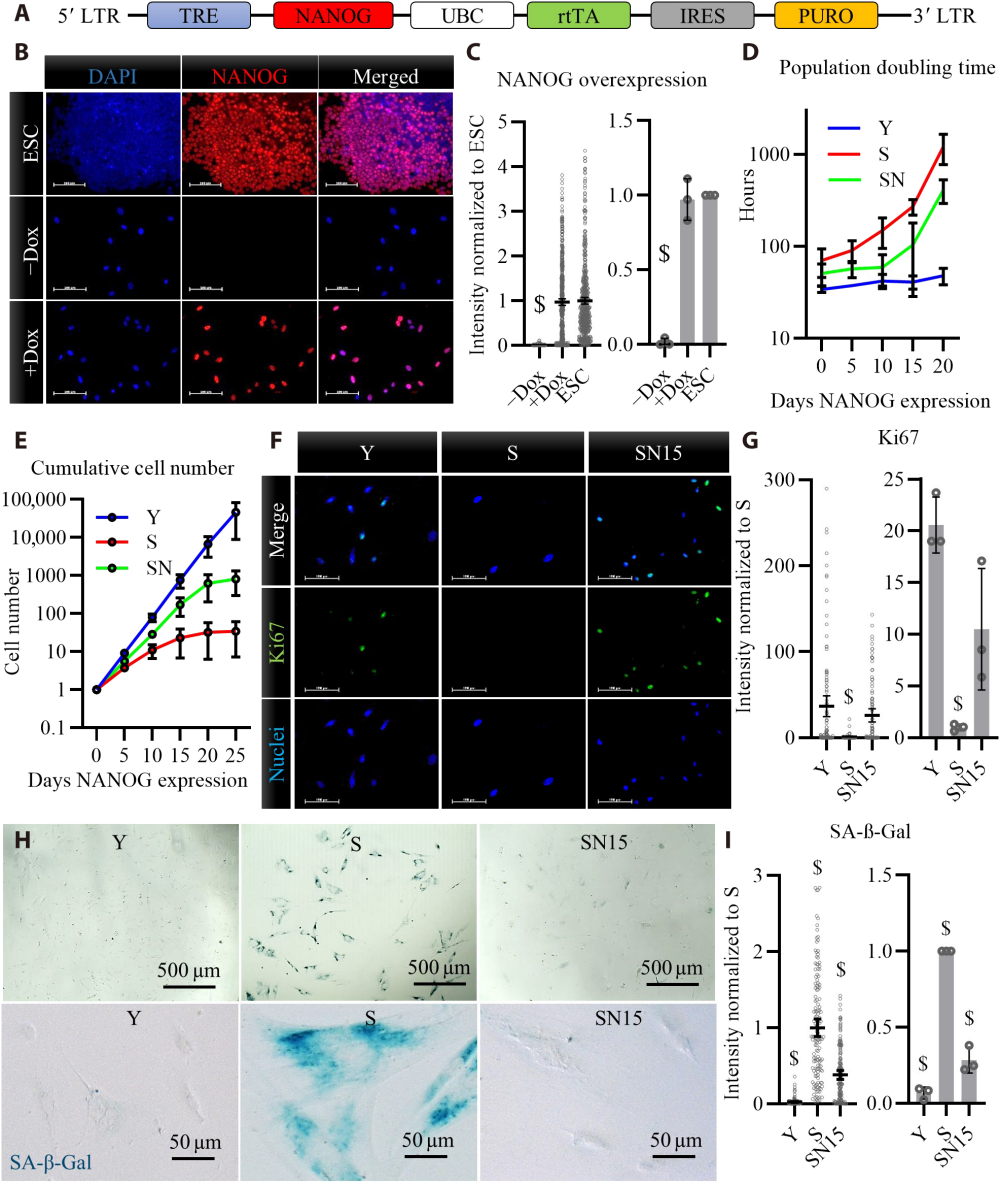
Overexpression of NANOG in human myoblasts after replicative senescence. (**A**) Schematic of the lentiviral vector encoding for NANOG. LTR, Long Terminal Repeat. (**B**) Immunostaining for NANOG expression and nuclear localization upon addition of doxycycline (Dox) in the medium of NANOG transduced human myoblasts and comparison to endogenous NANOG expression in embryonic stem cells (ESCs). Scale bars, 100 μm. DAPI, 4′,6-diamidino-2-phenylindole. (**C**) Quantification of fluorescence intensity in (B) reported as means ± 95% confidence interval (CI) for *n* = 500 cells for each condition in one representative experiment and means ± SD for three donors. (**D**) Population doubling time for human myoblasts at early passage young (Y), late passage senescent (S), and S myoblasts expressing NANOG (SN), *P* < 0.05 according to two-way analysis of variance (ANOVA) analysis. (**E**) Cumulative cell number in Y, S, or SN cells; data shown as means ± SD for *n* = 3 donors; *P* < 0.05 according to two-way ANOVA analysis. (**F** and **G**) Ki67 immunostaining and quantification for Y, S, and S myoblasts expressing NANOG for 15 days (SN15). Scale bars, 100 μm; data shown as means ± 95% CI for *n* = 100 cells for each condition in one representative experiment and means ± SD for three donors. (**H** and **I**) Senescence-associated β-galactosidase (SA-β-Gal) staining and quantification; data shown as means ± 95% CI for *n* = 100 cells for each condition in one representative experiment and means ± SD for three donors. $ denotes statistically significant as compared to all other samples.

We examined whether the pluripotency factor, NANOG, can reverse the hallmarks of cellular senescence in myogenic progenitors in vitro and in vivo. To this end, the embryonic transcription factor NANOG was expressed in human myoblasts using a lentiviral vector that encodes for NANOG under a tetracycline regulatable promoter (Fig. 1A). Upon addition of doxycycline (Dox) for 2 days, NANOG was expressed and localized in the nucleus (Fig. 1, B and C) at a level similar to endogenous NANOG in ESCs. To acquire senescent (S) cells in vitro, the young myoblasts (Y, passage 2) were cultured in the absence of Dox for 12 passages to reach senescence, i.e., a time equivalent to about 30 population doublings after which they lost their proliferation capacity as shown by the increased doubling time, decreased cumulative cell number, and decreased % Ki67^+^ cell nuclei (Fig. 1, D to G). Upon expression of NANOG in S cells for 5, 10, or 15 days (denoted as SN5, SN10, or SN15), the doubling time decreased and the cumulative cell number increased as compared to S (Fig. 1, D and E). Notably, the population doubling increased and the cumulative cell number plateaued after 20 to 25 days of NANOG expression, suggesting that NANOG did not induce myoblasts to proliferate indefinitely (Fig. 1E). Immunostaining for the proliferation marker, Ki67, further confirmed that NANOG expression induced S cells to enter the cell cycle (Fig. 1, F and G). NANOG expression also lowered the intensity of senescence-associated β-galactosidase (SA-β-Gal; Fig. 1, H and I), a well-known marker for cellular senescence.

### Genome-wide RNA sequencing and gene set enrichment analysis

**Fig. 2.**
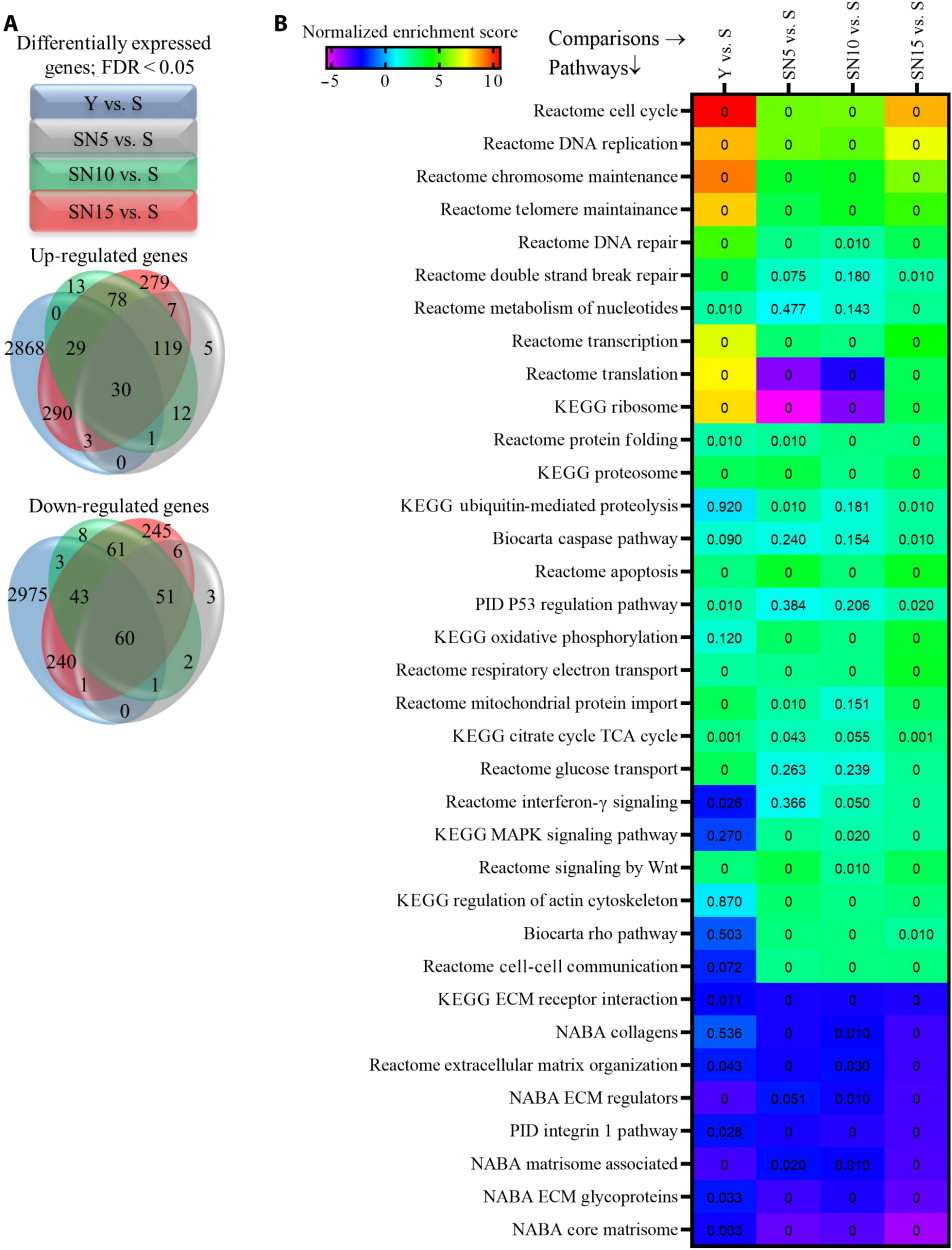
Genome-wide RNA-seq and GSEA. (**A**) Venn diagram of differentially expressed (DE) genes with false discovery rate (FDR) < 0.05 showing the distribution of overlapping or unique DE genes among different conditions. (**B**) A concise heatmap of representative pathways that are significantly enriched either at 5, 10, or 15 days after NANOG expression (FDR is written inside the cells; the FDR < 0.001 is depicted as 0). PID, Pathway Interaction Database.

We performed genome-wide transcriptional analysis using RNA sequencing (RNA-seq) to identify the differentially expressed (DE) genes between Y, S, and upon NANOG expression for 5, 10, or 15 days in S cells (the complete list of DE genes is shown in data S1). We assessed the distribution of overlapping or unique DE genes at different time points (Fig. 2A and data S2 and S3) and performed gene set enrichment analysis (GSEA) to identify the biological pathways that were associated with DE genes (data S4). NANOG expression significantly and dynamically modulated many pathways over time (data S5). The false discovery rate (FDR) and normalized enrichment score (NES) were calculated for each pathway to understand the statistical significance (FDR < 0.05) and the extent of up- or down-regulation (NES > 0 means up-regulated and NES < 0 means down-regulated). The complete list of up- or down-regulated pathways is shown in data S6, and the complete list of the overlapping and unique pathways at each time point is provided in data S7 and S8, respectively. Notably, expression of NANOG for 5, 10, or 15 days restored many pathways that were impaired by senescence such as cellular proliferation, genomic stability, proteostasis, and mitochondrial biogenesis, as well as pathways associated with senescent morphology such as regulation of actin cytoskeleton and the matrisome (Fig. 2B). Throughout the rest of this study, we confirm the amelioration of these senescence hallmarks in NANOG expressing myogenic progenitors in vitro and in vivo.

### NANOG restores nuclear integrity and heterochromatin modifications

**Fig. 3.**
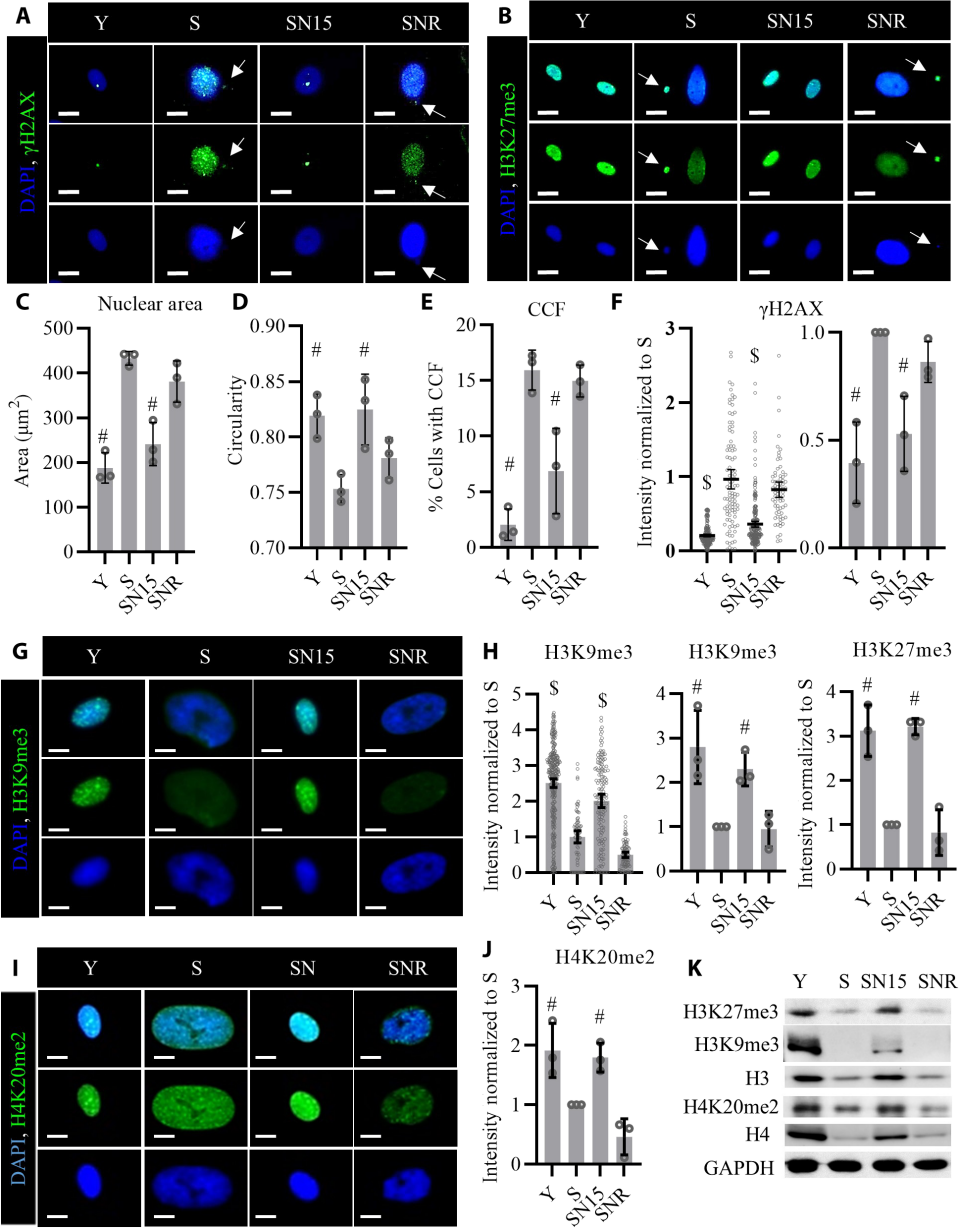
NANOG expression restores nuclear integrity and heterochromatin marks in senescent cells. (**A** and **B**) Immunostaining for phosphorylated form of histone H2AX (γH2AX) and H3K27me3. White arrows point to CCFs positive for γH2AX and H3K27me3. Scale bars, 20 μm. (**C** and **D**) Quantification of the nuclear area and nuclear circularity, data shown as means ± SD for three donors. (**E**) Quantification of percent cells positive for CCF. Data shown as means ± SD for three donors. (**F**) Quantification of γH2AX intensity per cell; data shown as means ± 95% CI for >100 cells for each condition in one representative experiment and means ± SD for three donors. (**G**) Immunostaining for heterochromatin mark H3K9me3. Scale bars, 10 μm. (**H**) Quantification of the H3K9me3 and H3K27me3 intensity. Data shown as means ± 95% CI for >100 cells for each condition in one representative experiment and means ± SD for three donors. (**I**) Immunostaining for the chromatin mark H4K20me2. Scale bars, 10 μm. (**J**) Quantification of H4K20me2 intensity. Data shown as means ± SD for three donors. (**K**) Western blotting analysis of the histone marks. GAPDH, glyceraldehyde-3-phosphate dehydrogenase. $ denotes *P* < 0.05 as compared to all other samples. # denotes *P* < 0.05 as compared to S.

Upon replicative senescence, the size of the cell nucleus is enlarged and the nuclear integrity is compromised with nuclear membrane deformities that ultimately result in cytoplasmic chromatin fragments (CCFs) (*3*, *5*). NANOG expression in S cells significantly decreased the nuclear size and increased nuclear circularity (Fig. 3, A to D). Furthermore, NANOG expression also reduced γH2AX- and H3K27me3-positive CCFs originating from the nucleus of S cells (Fig. 3, A, B, and E). In line with previous reports (*14*), we also observed decreased heterochromatin in S cells as shown by the immunostaining for heterochromatin marks H3K9me3 and H3K27me3. The heterochromatin foci were reestablished, and the levels of H3K9me3 and H3K27me3 (as shown by fluorescence intensity) were increased after 15 days of NANOG expression in S cells (Fig. 3, B, G, H, and K), which may be due to gradual transcriptional up-regulation of histones and histone methyl transferase Enhancer Of Zeste 2 Polycomb Repressive Complex 2 Subunit (EZH2) in SN cells (Fig. 3K and fig. S1). It is of note that the presence of NANOG was essential to maintain these epigenetic changes, because upon removal of Dox [pausing NANOG expression, SNR (SN15 cells after removal of NANOG for 2 weeks)], these heterochromatin marks decreased to the levels of S cells (Fig. 3, G, H, and K). We also observed improvements in the dimethylation of H4K20 (Fig. 3, I to K), which is essential for DNA repair and genomic stability (*28*). Moreover, the significant decrease in the phosphorylated form of histone variant H2AX (*5*) (γH2AX; Fig. 3, B and F) suggested lower levels of DNA damage in SN15 cells and prompted us to assess the prevalence of DNA double-strand breaks (DSBs) and the activation of DNA damage response (DDR) in SN cells. Treatment of control S cells (without NANOG transgene) with Dox had no effect on H3K9me3 and γH2AX levels, indicating that these effects were solely due to NANOG (fig. S2).

### NANOG reactivates DDR in senescent cells

**Fig. 4.**
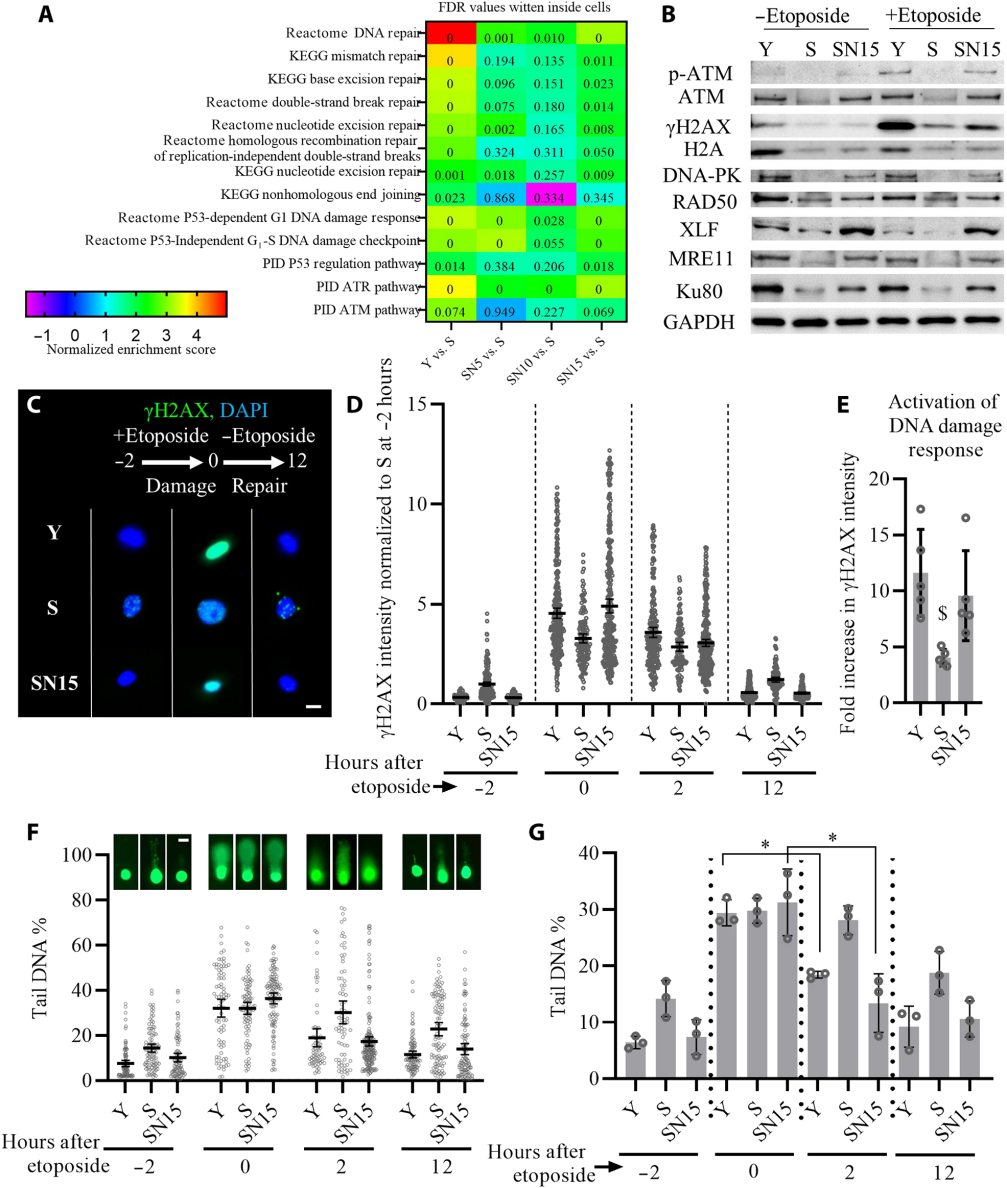
NANOG improves DNA repair and genomic stability in senescent myoblasts. (**A**) GSEA analysis of the DNA repair pathways altered by replicative senescence and NANOG expression for 5, 10, or 15 days. (**B**) Western blotting analysis of DNA repair proteins as well as phosphorylation of ATM and histone variant H2AX (γH2AX) in response to 2-hour treatment with the genotoxic chemical etoposide. (**C**) Phosphorylation of histone H2AX (γH2AX) before etoposide treatment (−2 hours) or at 0 and 12 hours after etoposide treatment. Scale bar, 20 μm. (**D**) Quantification of γH2AX intensity before etoposide treatment (−2 hours) or at 0, 2, and 12 hours after etoposide treatment; data shown as means ± 95% CI for >100 cells in one representative experiment of three independent experiments. (**E**) Fold increase in the intensity of γH2AX for each condition calculated by dividing the intensity of γH2AX after etoposide treatment to the intensity of γH2AX before the treatment, as a metric for DDR activation. Data shown as means ± SD of five independent experiments, and $ denotes *P* < 0.05 as compared to all other samples. (**F** and **G**) Representative images of single-cell gel electrophoresis assay (Comet assay) quantification of % tail DNA before (−2 hours) or at 0, 2, and 12 hours after etoposide treatment; data shown as means ± 95% CI for >100 cells per condition in one representative experiment (F, scale bar, 40 μm) and means ± SD for three donors (G). * denotes *P* < 0.05 in the comparison.

A major driver of cellular senescence is gradual accumulation of genetic damage that results in genomic instability (*3*, *29*). GSEA analysis of RNA-seq data suggested replicative senescence down-regulated, and NANOG restored the pathways related to DDR to repair DNA DSBs [through ataxia telangiectasia mutated (ATM)] and single-strand breaks [through ataxia telangiectasia and Rad3 related (ATR)]. Multiple DNA repair pathways (i.e., mismatch repair, nucleotide excision repair, interstrand cross-link recognition, and homologous recombination) as well as p53-dependent G_1_ DDR and the p53 pathway were restored by NANOG (Fig. 4A). Western blotting further confirmed that NANOG expression not only restored the levels of DNA repair proteins in S cells but also recovered the phosphorylation of ATM and γH2AX in response to the acute DNA damage (Fig. 4B). Acute DNA damage was induced by 2-hour treatment with the genotoxic chemical etoposide (10 μg/ml), which produces DNA breaks by inhibiting the topoisomerase II complex DNA religation step (*30*).

Upon induction of DNA damage, DDR is mediated through γH2AX that recruits and assembles DNA repair proteins to the site of damage (*31*). To assess the kinetics and efficiency of DDR upon induction of DSB, we measured the level of γH2AX via immunostaining (Fig. 4C) and % tail DNA via Comet assay (Fig. 4F) before and after etoposide treatment. Before etoposide treatment (−2 hours), S cells showed higher level of γH2AX and % tail DNA, which was significantly reduced by NANOG (Fig. 4, C, D, F, and G), suggesting less DSB in SN15 cells. However, upon etoposide treatment (0 hours), Y and SN15 cells showed higher levels of γH2AX as compared to S (Fig. 4, C and D) despite similar levels of DSB (Fig. 4, F and G). The increase of γH2AX upon DNA damage was lower in S cells but higher in Y and SN15 cells (Fig. 4E), suggesting higher activity of DDR in Y and SN15 cells. After removal of etoposide for 2 hours, Y and SN15 cells exhibited a faster decrease in γH2AX and % tail DNA (Fig. 4, C, D, F, and G), suggesting a more efficient DNA repair in Y and SN15 cells. After 12 hours, γH2AX and % tail DNA was significantly decreased in all conditions, with S cells showing higher levels of tail DNA% and γH2AX, similar to what was observed before etoposide treatment (Fig. 4, C, D, F, and G).

### NANOG promotes proteolysis and autophagy and decreases the size of senescent cells

**Fig. 5.**
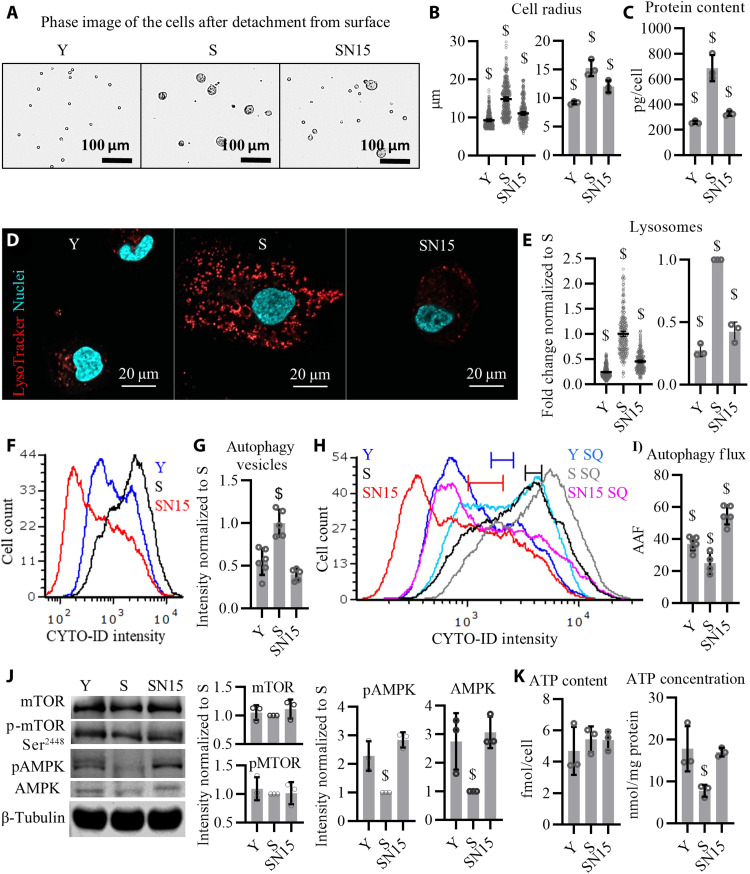
NANOG expression restores autophagy. (**A**) Phase images of the myoblasts detached from the surface. (**B**) Quantification of cell radius; data shown as means ± 95% CI for 300 cells per condition in one representative experiment and means ± SD for three donors. (**C**) Total intracellular protein content; data shown as means ± SD for three donors. (**D**) LysoTracker staining showing lysosomes. (**E**) Quantification of LysoTracker stain; data shown as means ± 95% CI for 300 cells per condition in one representative experiment and means ± SD for three donors. (**F**) Flow cytometry histogram of cells stained with CYTO-ID, a dye that binds to autophagy vesicles. (**G**) Quantification of the mean fluorescence intensity (MFI); data shown as means ± SD of five independent experiments. (**H**) Flow cytometry histogram of cells stained with CYTO-ID before or after starvation with chloroquine (SQ) treatment. (**I**) Autophagy activity factor (AAF % = 100 × (MFI_SQ_ − MFI_control_)/MFI_SQ_), a metric of autophagy flux; data shown as means ± SD for *n* = 5 independent experiments. (**J**) Western blotting analysis for quantifying the total protein and phosphorylation of mTOR (S2448) and AMPKα (T172). β-Tubulin serves as a housekeeping protein for loading control. (**K**) Total cellular ATP content reported as femtomole per cell and ATP concentration reported as nanomole per milligram of protein; data shown as means ± SD for three donors. $ denotes *P* < 0.05 as compared to all other samples.

GSEA analysis suggested enrichment of proteasome and ubiquitin mediated proteolysis by NANOG (fig. S3, A and B). At the same time, NANOG decreased protein synthesis and polypeptide elongation pathways in S cells transiently (at SN5 and SN10) and enriched protein-folding processes (fig. S3, C to E). In agreement, protein content and cell size, which were significantly increased in S cells, gradually decreased upon NANOG expression (Fig. 5, A to C, and fig. S3, F and G). It is of note that the presence of NANOG was essential to the maintenance of the small cell size, as upon removal of Dox (SNR condition), the cells regained their senescent morphology within 14 days (fig. S3, F and G).

The decreased expression of SA-β-Gal (Fig. 1, H and I), which is lysosomal β-d-galactosidase (*32*), prompted us to assess the process of autophagy. Slow rate of autophagy and accumulation of autophagy vesicles and lysosomes are characteristics of senescent phenotype (*33*–*35*). To this end, we visualized lysosomal trafficking using LysoTracker (Fig. 5, D and E), a hydrophobic weak base that accumulates in lysosomes (*36*). We also quantified autophagy vesicles by performing flow cytometry for CYTO-ID (Fig. 5, F and G), a cationic amphiphilic tracer dye that fluoresces only upon entering into autophagy vesicles but not lysosomes (*37*). The levels of lysosomes and autophagy vesicles were much higher in S cells but decreased in SN15 to the level of Y cells (Fig. 5, D to G).

To measure autophagy flux, we stimulated autophagy by starving the cells for 1.5 hours, while inhibiting autophagosome-lysosome fusion by chloroquine, leading to accumulation of autophagosomes [SQ (starvation+chloroquine) condition]. CYTO-ID staining before and after SQ treatment showed a significantly larger shift in fluorescence intensity for Y and SN15 as compared to S cells. As a result, the autophagy activity factor (AAF) was significantly reduced in S cells but up-regulated by NANOG to even higher levels than that of Y cells (Fig. 5, H and I). We further assessed autophagosome formation in Y, S, and SN15 cells in response to SQ treatment by performing Western blots for the autophagosome markers Microtubule-associated protein 1A/1B-light chain 3 (LC3-II) and observed a higher accumulation of LC3-II (SQ-Basal) in Y and SN15 cells, indicating greater capacity in forming autophagosomes (fig. S3H). Such increased autophagy flux might have cleared misfolded proteins and dysfunctional organelles in S cells, contributing to reduced size and total protein content per cell (Fig. 5, A to C).

Despite such marked functional changes, RNA-seq analysis did not show significant up-regulation of the autophagy pathway (fig. S3I), and the levels autophagy proteins [Beclin-1, Autophagy Related 12 (ATG12), ATG7, and ATG5] remained unchanged between Y, S, and SN15 cells (fig. S3, J and K). Therefore, we hypothesized that increased autophagy might not be due to transcriptional up-regulation but activation of pathways driving the autophagy machinery. To address this hypothesis, we measured the phosphorylation of Mammalian target of rapamycin (mTOR) at Ser^2448^, which is attributed to the formation of mTOR complex 1 (mTORC1) and is known to negatively regulate autophagy (*38*); and the phosphorylation of adenosine monophosphate-activated protein kinase α (AMPKα) at Thr^172^, which activates AMPK and positively regulates autophagy (*39*). Although there was no difference in phosphorylation of mTOR (p-mTOR) at Ser^2448^ among the three conditions (Fig. 5J), immunoblots revealed that both phosphorylation of AMPKα (pAMPKα) and total level of AMPKα decreased in S cells but were restored upon expression of NANOG (Fig. 5J). Besides autophagy, AMPK activity has been associated with energy homeostasis and adenosine 5′-triphosphate (ATP) concentration. Although because of the large size of S cells, the total cellular ATP (nanomole of ATP per cell) was not different between Y, S, and SN15 cells, the ATP concentration (nanomole of ATP per milligram of protein) was significantly lower in S cells and restored to Y in SN15 cells (Fig. 5K).

### NANOG expression improves mitochondrial health

**Fig. 6.**
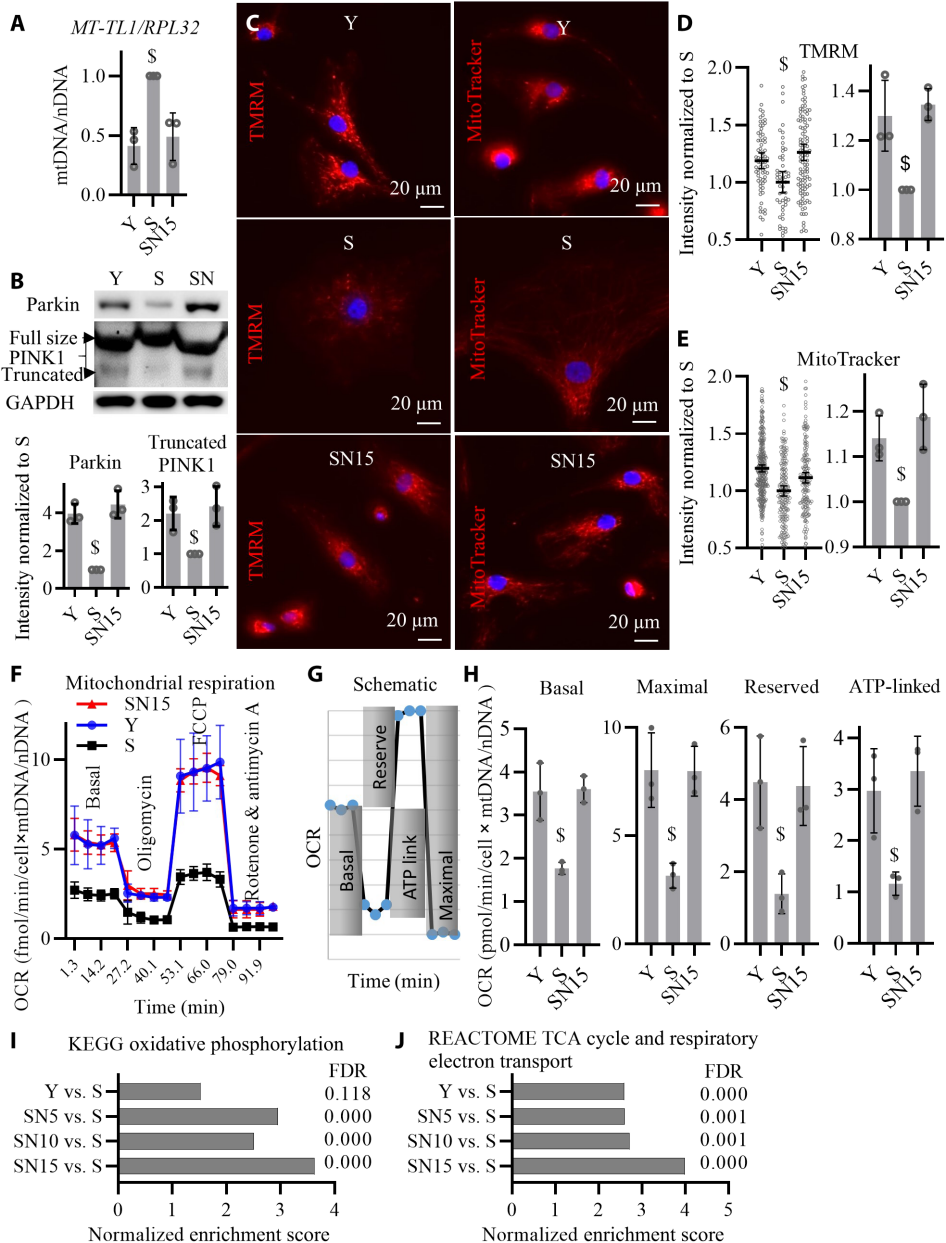
NANOG improves mitochondrial health. (**A**) Quantitative real-time PCR quantification of mtDNA relative to nDNA (mtDNA/nDNA) as measured by primers specific to mitochondrial gene *MT-TL1* and nuclear gene *RPL32*. Data shown as means ± SD for three donors. (**B**) Western blotting quantification of Parkin and PINK1 proteins. (**C**) Representative images of tetramethylrhodamine methyl ester (TMRM) and MitoTracker live stains corresponding to mitochondrial Δψ. (**D** and **E**) Quantification of TMRM and MitoTracker intensity per cell; data shown as means ± 95% CI for 200 to 300 cells per condition in one representative experiment and means ± SD for three donors. (**F**) Measurements of oxygen consumption rate (OCR) using Seahorse extracellular flux analyzer. (**G**) Schematic for the measurements of basal, maximal, reserved, and ATP-linked respiration rates. (**H**) Calculations of the basal, maximal, reserved, and ATP-linked respiration rates; data shown as means ± SD for three donors. (**I** and **J**) GSEA bioinformatics pathway analysis of RNA-seq shows enrichment of oxidative phosphorylation pathway as well as TCA cycle and respiratory electron transport pathway by NANOG expression for 5, 10, and 15 days in senescent cells. Data shown as means and FDR values for three donors. $ denotes *P* < 0.05 as compared to all other samples.

A hallmark of aging and cellular senescence is the accumulation of dysfunctional mitochondria due to inactive autophagy that ultimately alters cellular energetics (*3*, *5*). The increase in the autophagy flux, AMPK activation, and restoration of ATP levels by NANOG (Fig. 5) prompted us to evaluate mitochondrial function. To this end, we measured cellular mitochondrial content by quantitative polymerase chain reaction (qPCR) quantification of mitochondrial DNA over nuclear DNA (mtDNA/nDNA) and the levels of mitochondrial quality control proteins, Parkin and PINK. PINK1 is imported into the inner membrane of healthy mitochondria where it is cleaved and targeted for degradation; while, in damaged mitochondria, PINK1 accumulates on the outer mitochondrial membrane recruiting the ubiquitin ligase Parkin that induces mitophagy (*40*). Our results showed that S cells had higher levels of mtDNA/nDNA (Fig. 6A) but lower levels of Parkin and truncated PINK1 (Fig. 6B), suggesting accumulation of damaged mitochondria in S cells due to diminished mitophagy. Expression of NANOG for 15 days decreased mtDNA/nDNA and up-regulated the levels of Parkin and truncated PINK1 to the levels of Y, suggesting restoration of mitophagy that eliminated dysfunctional mitochondria in SN15 cells.

Mitochondrial membrane potential (ΔΨ), an indicator of mitochondrial function, was assessed by the fluorescence intensity of tetramethylrhodamine methyl ester (TMRM) and MitoTracker Red CMXRos fluorescent probes that accumulate in mitochondria in proportion to ΔΨ (*41*) and showed that Y and SN15 cells have higher ΔΨ as compared to S (Fig. 6, C to E).

To assess mitochondrial respiration, we used the Seahorse XF Analyzer to measure the oxygen consumption rate (OCR) after treating Y, S, and SN15 cells with oligomycin (mitochondrial complex V inhibitor that decreases electron flow in electron transport chain), carbonylcyanide-*p*-trifluoromethoxyphenylhydrazone (FCCP) (the uncoupling agent that collapses the proton gradient and maximizes electron flow), and a mixture of rotenone and antimycin A (complex I and complex III blockers that shut down mitochondria) (Fig. 6F). We normalized the OCR values to the cellular mitochondrial content and calculated the basal, maximal, reserved, and ATP-linked respiration rates as shown in Fig. 6G and found that they were all down-regulated in S and restored in SN15 cells (Fig. 6H), suggesting that NANOG increased mitochondrial respiration and oxidative phosphorylation. GSEA analysis of RNA-seq data also revealed that the pathways of oxidative phosphorylation, tricarboxylic acid (TCA) cycle, and respiratory electron transport were significantly up-regulated by expression of NANOG for 5, 10, and 15 days in S cells (Fig. 6, I and J, and fig. S4). Addition of Dox in control S cells (without NANOG transgene) had no effect on mitochondrial membrane potential or mitochondria respiration (fig. S5).

### AMPK activation is necessary for restoration of autophagy and mitochondrial function by NANOG

**Fig. 7.**
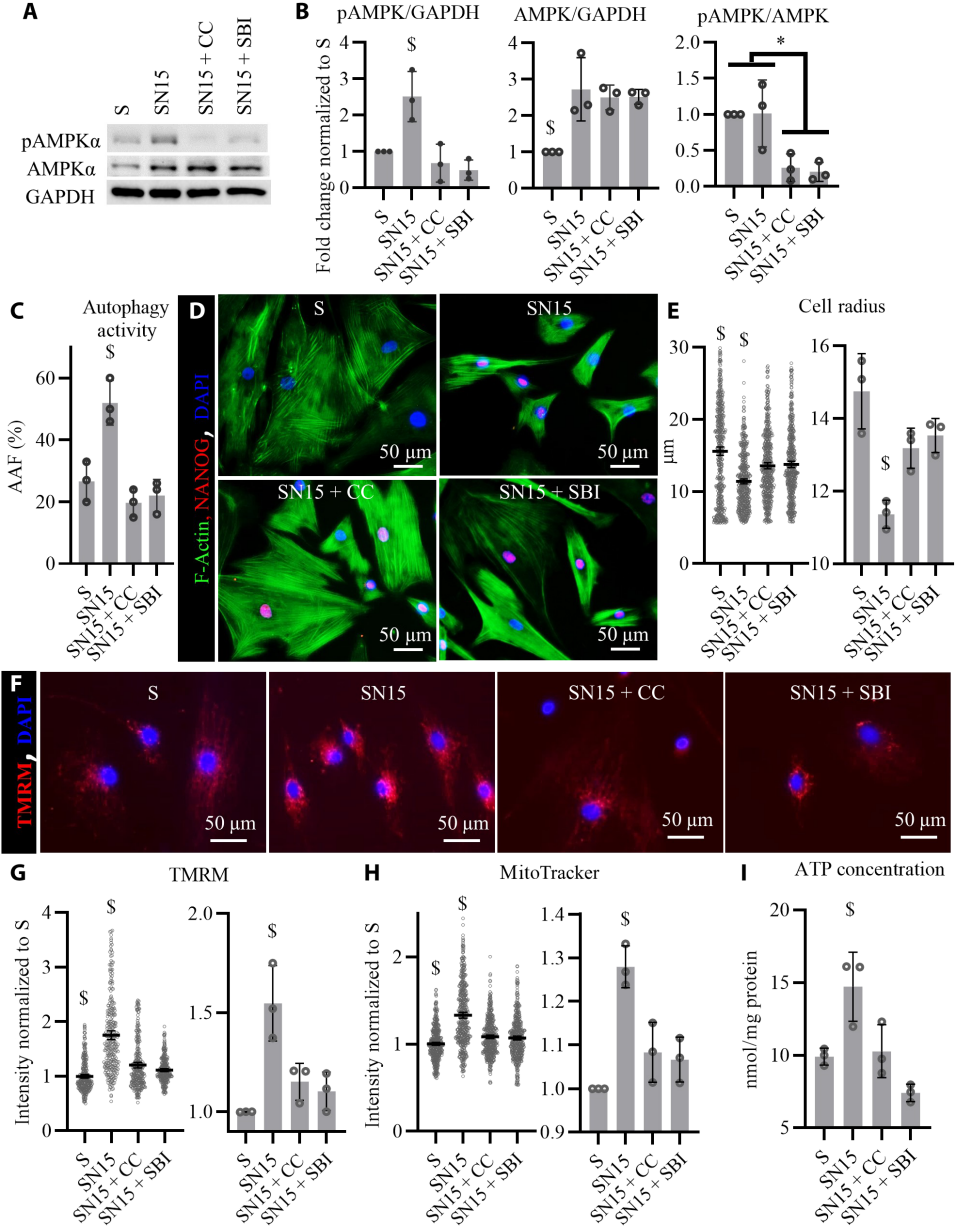
Inhibition of AMPK abrogated the up-regulation of autophagy and mitochondrial function by NANOG. AMPKα was inhibited along with NANOG expression in senescent myoblasts for 15 days. (**A**) Western blots against pAMPKα (T172) and total AMPKα, having GAPDH as a loading control. (**B**) Quantification of Western blots showed that NANOG expression significantly up-regulated the pAMKP/GAPDH by increasing AMPK/GAPDH levels, while addition of compound C (CC; 1 μg/ml) or SBI decreases the phosphorylated form of AMPKα without changing the total levels of AMPKα. Data shown as means ± SD for three independent Western blots. (**C**) Calculation of AAF suggests that AMPK activation is essential to the up-regulation of autophagy in SN cells. Data shown as means ± SD for three independent repeats. (**D**) Representative images of F-actin and NANOG immunostaining showing changes in cellular size. (**E**) Quantification of cell radius showing AMPKα significantly affects the decrease in cellular size by NANOG expression. Data shown as means ± 95% CI for 400 cells per condition in one representative experiment and means ± SD for three donors. (**F**) Representative images of TMRM staining corresponding to Δψ. (**G** and **H**) Quantification of TMRM and MitoTracker intensity as a measure of mitochondrial membrane potential. Data shown as means ± 95% CI for 300 cells per condition in one representative experiment and means ± SD for three donors. (**I**) Intercellular ATP concentration suggests that AMPK activation is essential to the effects of NANOG on cellular energetics. Data shown as means ± SD for three donors. $ denotes *P* < 0.05 as compared to all other samples. * denotes *P* < 0.05 between the two groups.

Increased levels of total AMPKα and pAMPKα at Thr^172^ suggested that activation of AMPK in SN cells might be necessary for the enhancement of autophagy and mitochondrial function by NANOG. To test this hypothesis, we inhibited AMPK in SN cells by addition of compound C (CC; 1 μg/ml) or SBI-0206965 (SBI), which inhibit AMPK by occupying a pocket that partially overlaps with the ATP active site in AMPKα subunit (*42*, *43*). Western blots for AMPKα and pAMPKα (Fig. 7A) confirmed that SBI and CC reduced the levels of pAMPKα to the control levels without changing the total AMPKα levels (Fig. 7B). Inhibition of AMPKα phosphorylation in SN cells abrogated the effects of NANOG on autophagy (Fig. 7C) and increased cellular size significantly (Fig. 7, D and E). AMPK inhibition also diminished the effects of NANOG on mitochondrial membrane potential as measured by the intensity of TMRM and MitoTracker (Fig. 7, F to H) and reduced the SN15 intracellular ATP concentration to the level of S cells (Fig. 7I).

### Development of fast aging mice that express NANOG upon Dox administration

**Fig. 8.**
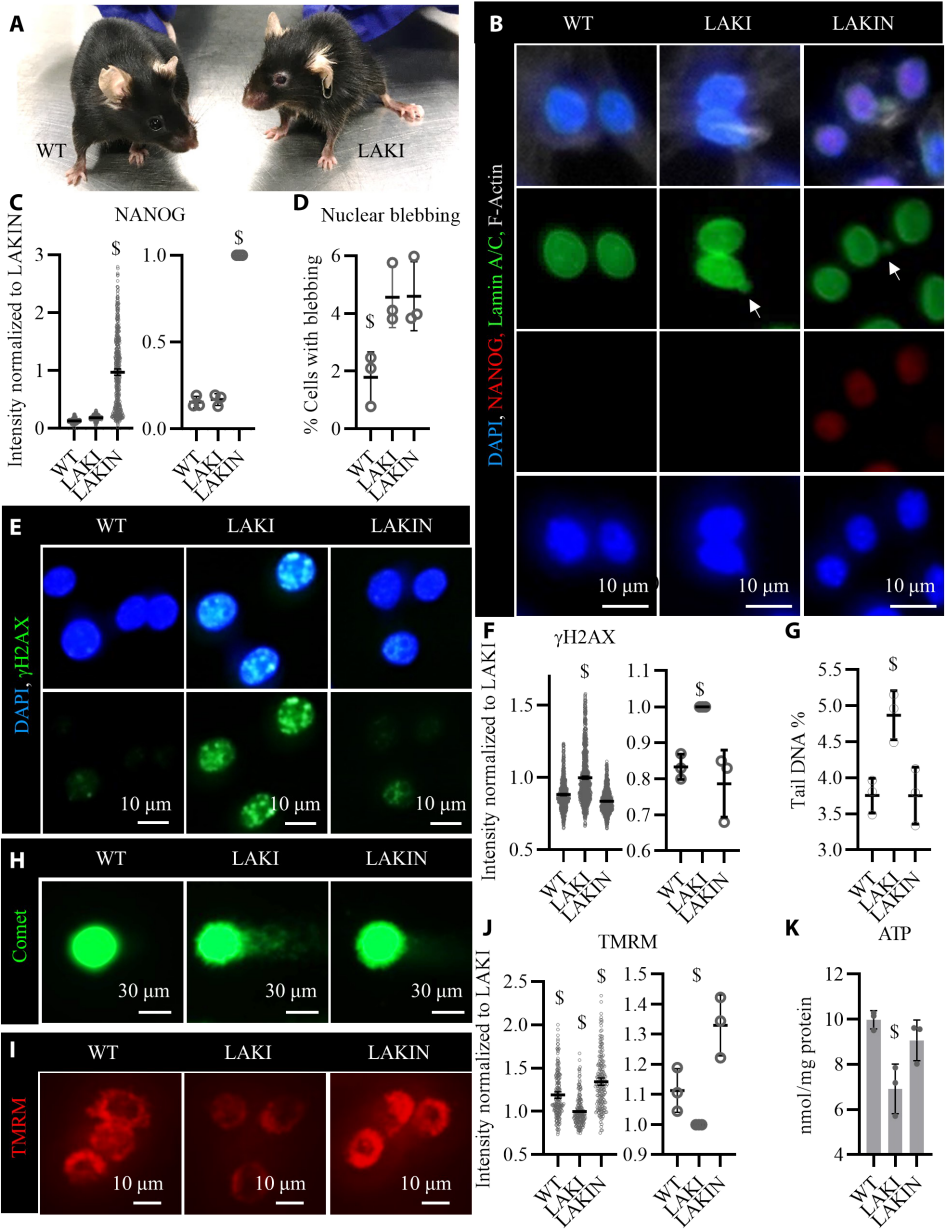
Expression of NANOG reverses cellular senescence in myoblasts isolated from progeria mice. (**A**) Representative picture of a heterozygous LAKI progeria mouse (*Lmna^G609G/+^*) and his WT sibling (*Lmna^+/+^*) both at the age of 10 months. (**B**) Immunostaining for NANOG and lamin A/C confirmed the expression of NANOG upon addition of Dox (1 μg/ml) in the medium (LAKIN) and the presence of nuclear blebs (white arrows) in both LAKI and LAKIN condition due to LMNA mutation. (**C**) Quantification of NANOG expression. Data shown as means ± 95% CI for 500 cells in one representative experiment and means ± SD for three independent experiments. (**D**) Quantification of the percent cells with nuclear blebs. Data shows the means ± SD for three independent experiments. (**E**) Immunostaining for phosphorylated form of histone H2AX (γH2AX). (**F**) Quantification of γH2AX intensity. Data shown as means ± 95% CI for 500 cells per condition in one representative experiment and means ± SD for three independent experiments. (**G**) Quantification of the DNA DSBs measured as the tail DNA percent in single-cell electrophoresis (Comet) assay. Data shown as means ± SD for three independent experiments. (**H**) Representative image of comets for each condition. (**I**) Representative image of the TMRM fluorescence intensity, a measure of mitochondrial membrane potential. (**J**) Quantification of TMRM intensity. Data shown as means ± 95% CI for 200 cells per condition in one representative experiment and means ± SD for three independent experiments. (**K**) Intracellular ATP concentration as a metric for cellular energetics. Data shown as means ± SD for three independent experiments. $ denotes *P* < 0.05 as compared to all other samples. Photo credit: Aref Shahini, University at Buffalo, The State University of New York.

Progeroid syndromes are due to genetic disorders affecting nuclear envelop and DNA repair mechanisms and they mimic physiological ageing (*44*). The LAKI model of progeria results in accumulation of the truncated form of LMNA (progerin) (*12*), which normally accumulates over time with aging and induces nuclear defects in old individuals (*13*). To assess the effects of NANOG expression in regenerating muscle in vivo, we crossed LAKI and Tet-On-NANOG mice to obtain progeny with fast-aging phenotype capable of NANOG expression upon Dox administration (LAKIN). The animals used in this study expressed NANOG under the tetracycline inducible promoter (TetO) from the Col1 locus and the reverse tetracycline transactivator (rtTA) from the ROSA26 locus. They were heterozygous for the LMNA mutation (*Col1a1^tetO-NANOG/+^;ROSA26^rtTA/+^;Lmna^G609G/+^*), which induced premature aging at the age of 10 months (Fig. 8A).

To assess the effects of NANOG on the myogenic progenitors of these fast aging mice, we isolated myoblasts from their skeletal muscle according to our established protocol (*45*) and subsequently expressed NANOG in the LAKI myoblasts by addition Dox (1 μg/ml) in the medium (Fig. 8, B and C). Although NANOG expression did not decrease the nuclear blebs induced by *Lmna* mutation in LAKI myoblasts (Fig. 8, B and D), it significantly decreased the incidence of DNA damage as measured by the intensity of γH2AX (Fig. 8, E and F) and comet tail DNA % (Fig. 8, G and H). Furthermore, ectopic expression of NANOG in LAKI myoblasts restored mitochondrial membrane potential as measured by the intensity of TMRM (Fig. 8, I and J) and cellular energetics as measured by the intracellular ATP concentration (Fig. 8K). These results suggested that ectopic expression of NANOG in the skeletal muscle cells of the fast-aging LAKI mice might ameliorate the effects of cellular senescence and prompted us to examine whether NANOG affected the myogenic progenitors in skeletal muscle in vivo.

### NANOG expression restores the pool of myogenic progenitors and induce myofiber formation in vivo

**Fig. 9.**
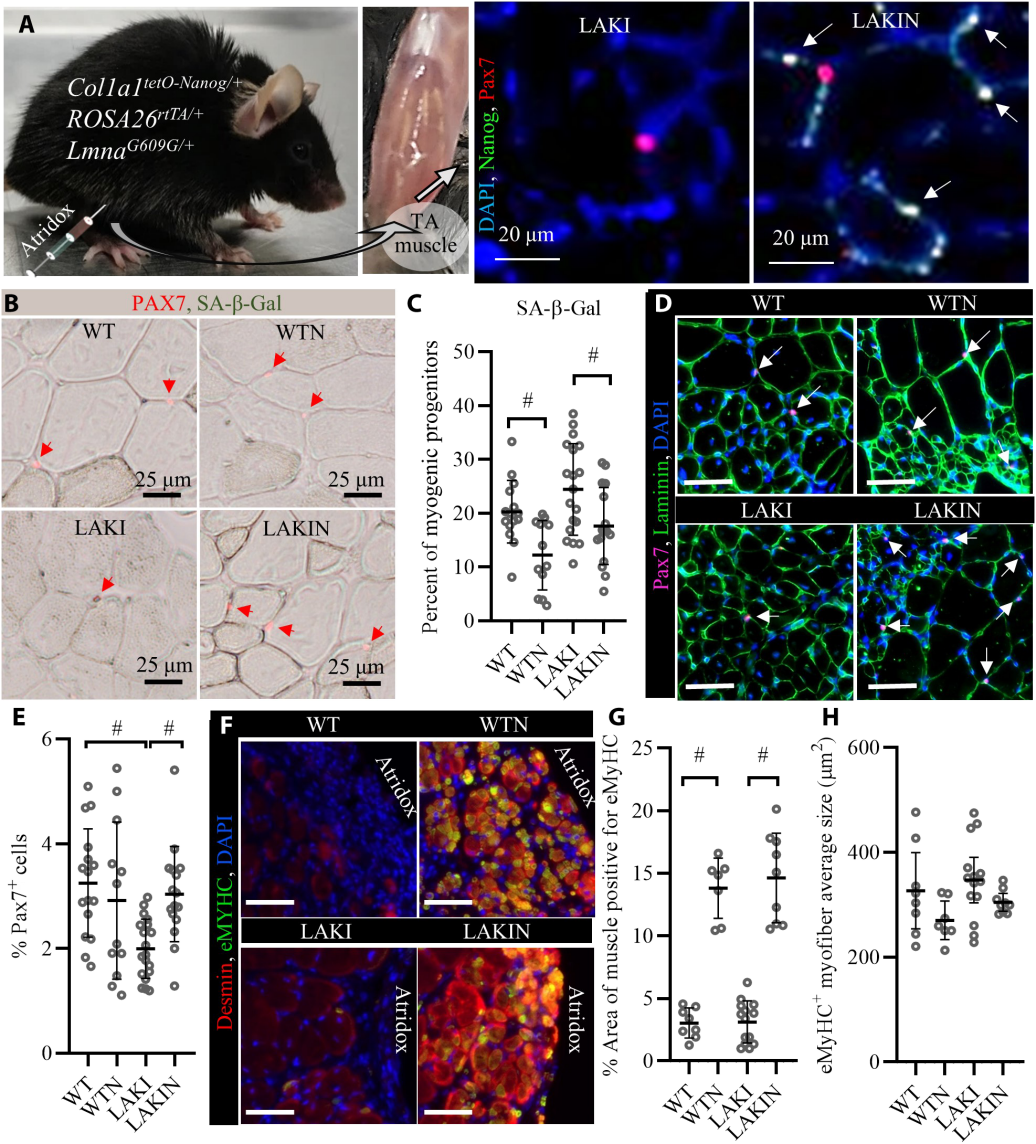
In vivo muscle regeneration upon NANOG expression. (**A**) Schematic of the experimental setup for NANOG expression in vivo using Atridox Dox delivery gel and immunostaining for Nanog and Pax7 to confirm NANOG expression in myogenic progenitors after Atridox injection (arrows point to PAX7^+^ NANOG^+^ myogenic progenitors). (**B** and **C**) Costaining for Pax7 and SA-β-Gal shows a decrease in the fraction of SA-β-Gal–positive cells by NANOG expression. Data shown as means ± SD for each cohort of animals (each bubble represents a mouse with 100 to 200 Pax7^+^ cell analyzed per animal; WT, *n* = 16; WTN, *n* = 12; LAKI, *n* = 19; LAKIN, *n* = 16). (**D**) Immunostaining for Pax7 and laminin shows increased number of Pax7^+^ myogenic progenitors and centrally nucleated myofibers at the site of Atridox injection. Scale bars, 100 μm. (**E**) Quantification of % Pax7^+^ cells. Data shown as means ± SD for each cohort of animals (each bubble represents a mouse with 10^4^ to 10^5^ cells analyzed per animal; WT, *n*= 16; WTN, *n* = 12; LAKI, *n* = 19; LAKIN, *n* = 16). (**F**) Immunostaining for Desmin (red) and eMyHC (green) shows the presence of small fibers with high intensity of Desmin and positive for eMyHC at the site of Atridox injection. Scale bars, 200 μm. (**G**) Quantification of the % area positive for eMyHC^+^ myofibers. Data shown as means ± SD for each cohort of animals (each bubble represents a mouse with 0.2 to 2 mm^2^ analyzed per animal; WT, *n* = 8; WTN, *n* = 7; LAKI, *n* = 14; LAKIN, *n* = 9). (**H**) Average size of eMyHC^+^ myofibers for each animal. Data shown as means ± SD for each cohort of animals (each bubble represents a mouse with 10 to 1000 eMyHC^+^ myofibers analyzed per animal; WT, *n* = 8; WTN, *n* = 7; LAKI, *n* = 14; LAKIN, *n* = 9). # denotes *P* < 0.05. Photo credit: Aref Shahini, University at Buffalo, The State University of New York.

To avoid systemic effects (*23*, *24*), NANOG was expressed locally in the tibialis anterior (TA) muscle by intramuscular injection of a Dox-containing hydrogel (Atridox) that enables sustained release of Dox in the surrounding muscle over a period of 3 weeks (Fig. 9A and fig. S6) (*46*, *47*). Wild-type (WT) and transgenic (LAKI) mice expressing NANOG were denoted as wild type expressing NANOG (WTN) and LAKIN, respectively. The animals were sacrificed 3 or 5 weeks after Atridox injection, and their TA muscles were isolated and processed for further analysis. Immunostaining for NANOG and Pax7 (*48*) confirmed the expression of NANOG and the presence of myogenic progenitors at the site of Atridox injection (Fig. 9A). NANOG expression significantly decreased the percentage of SA-β-Gal^+^ among the Pax7^+^ myogenic progenitors (Fig. 9, B and C). It also increased the number of myogenic progenitors that were otherwise low in LAKI animals (Fig. 9, D and E). In this region, we observed an abundance of small myofibers (area ≈ 300 μm^2^) that were positive for embryonic myosin heavy chain (eMyHC) and desmin in WTN and LAKIN groups (Fig. 9, F to H), suggesting possible de novo myofiber formation in NANOG expressing muscle (*49*–*51*).

## DISCUSSION

**Fig. 10.**
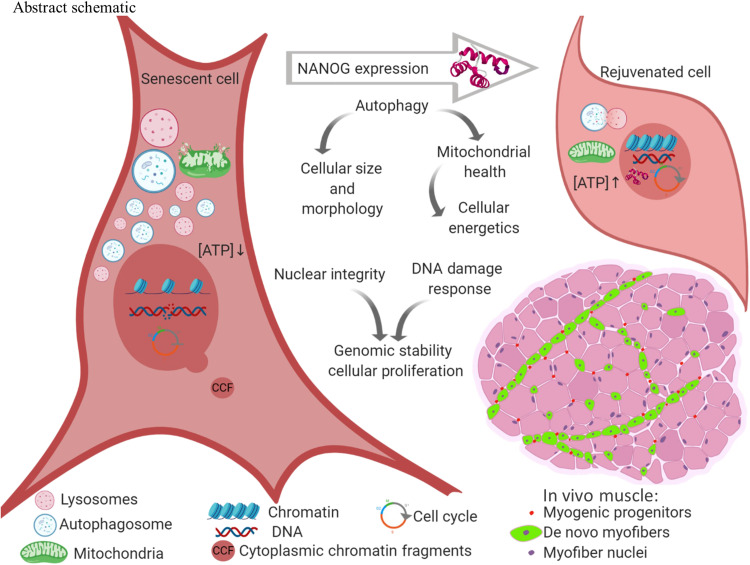
Schematic abstract, NANOG expression ameliorates the hallmarks of cellular senescence. Senescent cells expressing NANOG increased autophagy flux to remove dysfunctional mitochondria, reduce cellular size, and restore the ATP/protein content to the levels of their younger counterparts. NANOG also improved nuclear integrity, reduced the frequency of CCFs, and restored the heterochromatin marks H3K9me3 and H3K27me3. DDR was enhanced in NANOG expressing cells to repair DNA DSBs and allow reentry to the cell cycle. Notably, NANOG restored the pool of Pax7^+^ myogenic progenitors and induced eMyHC^+^ myofiber formation in a mouse model of premature aging.

Skeletal muscle regeneration depends on activation of satellite stem cells that remain quiescent until the need arises to enter the cell cycle and participate in myofiber regeneration. Previous research reported that 34 to 66% of these cells were positive for the transcription factor NANOG (*52*), and we showed that expression of NANOG preserved the myogenic potential of C2C12 myoblasts over multiple population doublings (*27*). Ectopic expression of NANOG in fibroblasts and mesenchymal stem cells was also shown to reverse many aspects of cellular senescence, including restoring their multipotency (*20*–*22*). Here, we show that ectopic expression of NANOG ameliorates several hallmarks of cellular senescence including proliferation, DDR, autophagy, and senescent morphology in myogenic progenitors (Fig. 10). In line with the literature (*53*, *54*), the hallmarks of cellular senescence were not observed in myogenic progenitors isolated from young or old adults at early passages. The senescence hallmarks were observed only after multiple rounds of population doubling in culture (*53*, *54*). Therefore, we adopted replicative senescence as the model of cellular senescence in vitro, where we saw all senescence hallmarks consistently present after 12 passages or 30 population doublings, irrespective of donor age or sex. We further confirmed improvements in the senescence hallmarks in vivo using a mouse model of Hutchinson-Gilford progeria syndrome, where expression of NANOG in the skeletal muscle restored the number of resident myogenic progenitors and induced formation of eMyHC^+^ myofibers.

Expression of NANOG in senescent myoblasts induced entry into the cell cycle, which may have been the result of improved genomic stability and DNA repair processes in NANOG-expressing senescent myoblasts, as evidenced by improved nuclear circularity and decreased incidence of CCFs (*55*). NANOG restored heterochromatins that are essential to maintaining nuclear architecture. The heterochromatin loss model of cellular aging proposes that the loss of heterochromatin in senescent cells induces the transcription of senescence-associated genes, as it loosens the heterochromatin domains that are normally compact and not accessible to transcription factors (*56*). Heterochromatins play an essential role in maintaining nuclear architecture, and decreased trimethylation of histone 3 at Lys^9^ and Lys^27^ (H3K9me3 and H3K27me3) was associated with senescence (*57*), while restoration of these epigenetic marks was shown to ameliorate the senescence phenotype (*14*, *57*, *58*). Similar to NANOG, partial reprogramming by ectopic expression of the four reprogramming factors OCT4, SOX2, KLF4, and MYC (OSKM) was reported to induce heterochromatin modifications albeit faster, within 12 hours of expression (*14*). The slower change of the H3K9me3 and H3K27me3 heterochromatin marks by NANOG may suggest an indirect mechanism, whereby NANOG affects epigenetic marks by up-regulating histones and histone methytransferases such as EZH2, which was up-regulated in NANOG expressing cells and is known to be essential for preserving H3K27 methylation (*58*) .

The recovery of chromatin mark H4K20me3 in SN myoblasts suggests restoration of DDR after NANOG expression (*28*). Accumulation of DNA damage is a hallmark of cellular senescence (*3*, *5*, *59*), and senescent cells exhibit an attenuated DDR (*60*). GSEA analysis of the DE genes from genome-wide RNA-seq suggested that NANOG expression could restore ATM, ATR, and the DNA repair pathways in senescent cells to intensify DDR and decrease DNA damage. In accordance with this, we observed significantly lower levels of DNA DSBs and γH2AX foci in SN cells. Furthermore, in response to the genotoxic chemical etoposide, SN cells showed a superior up-regulation of γH2AX that was summoned to repair DSB.

Increased cell size, higher mitochondrial content, and expression of SA-β-Gal are well-known marks of senescence (*5*). Notably, NANOG-expressing cells exhibited marked decreases in cell size, mitochondrial content, and SA-β-Gal expression as compared to control myoblasts. Because SA-β-Gal accumulates in lysosomes during senescence (*32*), we hypothesized that decreased SA-β-Gal activity might be attributed to decreased lysosomal content possibly due to enhanced autophagy flux. NANOG expression enhanced autophagy flux in senescent cells and increased mitochondrial quality control proteins, PINK1 and Parkin (*40*), to remove dysfunctional mitochondria via mitophagy and restore mitochondrial membrane potential, respiration, and energy production in senescent cells.

The mRNA or protein levels of autophagy related proteins were not significantly affected by replicative senescence or NANOG. This result agrees with a recent study showing that down-regulation of autophagy might not be due to decreased levels but rather decreased activity of autophagy regulators (*61*). Autophagy is negatively regulated by phosphorylation of mTOR at Ser^2448^ that is attributed to formation of mTORC1 (*38*) and positively regulated by phosphorylation of AMPKα subunit at Thr^172^ (*39*), by kinases such as liver kinase B1 (LKB1) and Calcium/Calmodulin-Dependent Protein Kinase Kinase 2, Beta (CamKKβ) upon decrease in ATP concentration (*62*). AMPK activation is essential for autophagosome maturation and lysosomal fusion (*63*), while loss of AMPK has been observed in cellular senescence (*64*). AMPK reactivation has been shown to improve autophagy, mitochondrial health, and energy homeostasis in senescent cells (*65*). Our results showed no change in the phosphorylation of mTOR at Ser^2448^. However, despite lower ATP concentrations in senescent cells, the phosphorylated levels of AMPKα were significantly down-regulated in senescent cells due to decrease in total AMPK, which was then restored by NANOG. Furthermore, inhibition of AMPK abrogated the effects of NANOG on autophagy, mitochondrial membrane potential, and intracellular ATP concentration, suggesting that the restoration of autophagy flux and cellular energetics might be due to reactivation of AMPK.

Most notably, NANOG decreased the prevalence of SA-β-Gal–positive myogenic progenitors and restored the number of these Pax7^+^ progenitors in the skeletal muscle of LAKI progeria mice in vivo. NANOG expression was induced in the TA muscle of 10 months old (*Col1a1^tetO-NANOG/+^;ROSA26^rtTA/+^;Lmna^G609G/+^*) progeria mice by intramuscular injection of Atridox, which polymerized in vivo and released Dox for a period of 2 to 3 weeks. Immunostaining showed that NANOG was expressed in all cells, including Pax7^+^ cells, in proximity to the Atridox injection sites but not in distant sites, possibly due to a combination of consumption and limited diffusion of Dox within the muscle. In this setting, NANOG expression did not alter the total number of Pax7^+^ myogenic progenitors in WT muscle (WTN versus WT) but significantly reduced the prevalence of SA-β-Gal^+^ myogenic progenitors in WT. In comparison to WT, LAKI muscles showed a significantly lower number of myogenic progenitors and higher percentage of SA-β-Gal^+^ cells, while NANOG expression restored the number of Pax7^+^ myogenic progenitors and decreased the prevalence of SA-β-Gal^+^ myogenic progenitors in LAKI muscles. Mutation of LMNA nuclear membrane protein in LAKI animals leads to accumulation of progerin that induces nuclear envelope abnormalities and genomic instability (*12*). Although NANOG did not correct the mutation or incidence of nuclear blebs, it restored the pool of myogenic progenitors, possibly by restoring genomic stability and DNA repair capacity of senescent LAKI myoblasts.

eMyHC is transiently expressed during development and disappears shortly after birth; it is also expressed in regenerating adult skeletal muscle between 3 days and 3 weeks after injury before it is replaced with adult fast or slow myosins (*51*). Because we isolated the TA muscle 3 to 5 weeks after Atridox injection, eMyHC was minimally present in WT and LAKI condition. However, in WTN and LAKIN condition, we observed more myofibers due to the abundance of small centrally nucleated myofibers (area ≈ 300 μm^2^) that were eMyHC positive, suggesting that NANOG might have induced de novo myofiber formation. Activation of myogenic progenitors is only part of the complex process of de novo myofiber formation that also requires extracellular matrix (ECM) remodeling to accommodate the formation of new fibers (*66*). Of note, our RNA-seq showed that NANOG significantly changed the expression of ECM genes and pathways associated with ECM regulation and organization. Nevertheless, more work including later time points with fully formed myotubes is required to establish whether these are functional new fibers that arose from activated satellite cells.

Myofibers in WNT and LAKIN muscles that were positive for eMyHC were also strongly positive for Desmin, possibly due to the essential role of this intermediate filament in the assembly of new myofibrils and sarcomeres in regenerating myofibers (*49*, *50*, *67*). Because NANOG expression interferes with myogenic differentiation (*27*), longer times following the release of Dox might be required for these myofibers to stop expressing NANOG and subsequently down-regulate eMyHC, increase in size, and fully mature. The presence of centrally nucleated myofibers close to the injection site was not due to NANOG expression as they also appeared in control tissues; they may be attributable to the injury created by the needle injection or the small pieces of Atridox polymer that had not degraded by the time the tissues were isolated.

In conclusion, NANOG ameliorated multiple senescence hallmarks including autophagy, energy homeostasis, genomic stability, nuclear integrity, and heterochromatin maintenance. Upon NANOG expression, senescent myogenic progenitors regained their capacity to proliferate in vitro and in vivo, without oncogenic transformation or reprogramming to the embryonic pluripotent state. Ultimately, understanding the mechanisms of NANOG’s actions may lead to discovery of druggable targets and facilitate the design of small molecules targeting signaling or metabolic pathways that mimic the anti-aging effects of NANOG.

## MATERIALS AND METHODS

### Cell culture

Human myoblasts from a 68-year-old male, a 75-year-old female, and a 25-year-old female were purchased from Cook Myosite Inc., Pittsburgh, PA, and ThermoFisher Scientific, Waltham, MA. The cells were expanded in growth medium (GM) composed of high-glucose Dulbecco’s modified Eagle’s medium (Gibco, Grand Island, NY) supplemented with 10% (v/v) fetal bovine serum (Atlanta Biologicals, Flowery Branch, GA), bovine serum albumin (BSA; 0.5 mg/ml; VWR, Radnor, PA), epidermal growth factor (10 ng/ml; Lonza, Allendale, NJ), basic fibroblast growth factor (1 ng/ml; Isokine, Kópavogur, Iceland), insulin (10 μg/ml; Sigma-Aldrich, St. Louis, MO), fetuin (50 μg/ml; Sigma-Aldrich), dexamethasone (0.2 μg/ml; VEDCO, Saint Joseph, MO), gentamycin (10 μg/ml; Gibco), 1% Antibiotic-Antimycotic (Gibco), and plasmocin prophylactic (2.5 μg/ml; Invivogen, San Diego, CA). To passage the cells, they were washed using phosphate-buffered saline (PBS), detached using 0.25% Trypsin-EDTA (Gibco), and centrifuged at 300*g* for 5 min. Tissue culture plates were coated with Matrigel before seeding the cells as we described previously (*45*). To block AMPK activity in the cells in culture, Compound C (CC; Cayman Chemical, Ann Arbor, MI, catalog no. 11967) or SBI-0206965 (SBI; Cayman Chemical, catalog no. 18477) were added in GM at a final concentration of 1 μg/ml.

Mouse myoblasts were isolated from male and female C57BL/6 WT mice or homozygous progeria mice capable of expressing NANOG upon Dox treatment (Col1a1 ^tetO-Nanog/+^, ROSA26^rtTA/+^, Lmna^G609G/G609G^) at the age of 3.5 months according to our previously established protocol (*45*). Homozygous progeria mice (G609G/G609G) show signs of aging after 3 months of age and die before they reach 4 months of age (*12*).

### Lentiviral transduction and ectopic expression of NANOG in human myoblasts

A tetracycline regulatable system was used to overexpress NANOG in myoblasts. Briefly, the cells were transduced with a lentivirus encoding for the reverse tetracycline-controlled transactivator (rtTA) and the selection marker puromycin *N*-acetyl transferase under the constitutively active ubiquitin promoter, as well as NANOG under the tetracycline response element (TRE-*NANOG*-UBC-rtTA-IRES-*PURO*). To generate this vector, first *hNANOG*-IRES-*PURO* gene was extracted from pSIN-*hNANOG*-IRES-*PURO* (Addgene, Cambridge, MA) and inserted into the tetracycline regulatable lentiviral vector, pNL-EGFP/TREPittdU3 (Addgene) to replace EGFP and create TRE-*hNANOG*-IRES-*PURO*. Subsequently, UBC-rtTA was extracted from FUdeltaGW-rtTA (Addgene) and inserted between *hNANOG* and IRES to create TRE-*NANOG*-UBC-rtTA-IRES-*PURO.* Recombinant lentiviruses were produced as described elsewhere (*68*), and human myoblasts were transduced with lentiviruses in GM supplemented with polybrene (8 μg/ml; Sigma-Aldrich) for 6 hours. Transduced cells were selected with puromycin at 1 μg/ml for 5 days. NANOG expression was induced by addition of Dox (Sigma-Aldrich) at 1 μg/ml in the medium, and a parallel culture without Dox treatment was used as control.

### Visualization of lysosomes and autophagy flux analysis

To visualize the lysosomes, the cells were incubated for 20 min with a fluorescent dye (LysoTracker Deep Red, ThermoFisher Scientific) that consists of a fluorophore linked to a weak base and selectively labels acidic organelles (lysosomes) inside the cells (*36*). Subsequently, the cells were washed with PBS, stained with Hoechst 33342 DNA dye (ThermoFisher Scientific) for 5 min, and imaged using a Zeiss Axio Observer Z1 microscope (LSM 510; Zeiss, Oberkochen, Germany) equipped with a high definition digital camera (ORCA-ER C4742-80; Hamamatsu, Bridgewater, NJ). For each sample, the intensity of the LysoTracker signal was quantified in 10 randomly chosen fields of view using the ImageJ software and divided by the total number of the cells in the same fields of view to calculate the LysoTracker intensity per cell.

To quantify the autophagic vesicles, the cells were stained with CYTO-ID Green autophagy dye according to the manufacturer’s protocol (Enzo Life Sciences, Farmingdale, NY, catalog no. ENZ-51031-0050). CYTO-ID is a cationic amphiphilic tracer dye that fluoresces upon compartmentalization with the lamellar membrane structures associated with pre-autophagosomes, autophagosomes, and autolysosomes but not lysosomes (*37*). CYTO-ID histograms were generated by flow cytometry analysis of 10,000 cells per sample using a BD LSRFortessa X-20 (BD Biosciences, Franklin Lakes, NJ) equipped with four lasers and 16 color analyzers. The results were analyzed using FCS Express 6 (De Novo software, Pasadena, CA). Autophagy flux was assessed by measuring the mean fluorescent intensity (MFI) of the cells before (Ctrl) or after starvation plus chloroquine treatment (SQ) for 1.5 hours, and the AAF was calculated on the basis of AAF = 100 × (MFI_SQ_ − MFI_Ctrl_)/MFI_SQ_. AAF is a dimensionless measure of the accumulation of autophagosomes normalized to the maximum autophagosome amount to eliminate the effects of cell size on autophagosome accumulation measurements (*37*).

### mtDNA content quantification

DNA was isolated using the QIAmp DNA Mini Kit (QIAGEN, Germantown, MD catalog no. 51304) according to the manufacturer’s instructions. Quantitative real-time PCR was performed using the SYBR Green Kit (Bio-Rad, Hercules, CA, catalog no. 172-5120) with 25 ng of DNA used per reaction. mtDNA was quantified using the following human primers for mitochondrially encoded tRNA^Leu (UUR)^ gene [MT-TL1, 5′-CACCCAAGAACAGGGTTTGT-3′ (forward primer) and 5′-TGGCCATGGGTATGTTGTTA-3′ (reverse primer)], and nDNA was quantified using the following human primers for ribosomal protein L32 gene [(forward primer) 5′-AGCGTAACTGGCGGAAAC-3′ and (reverse primer) 5′-CGTTGTGGACCAGGAACTTC-3′], respectively. Both mtDNA and nDNA threshold cycle (CT) average values were obtained, and the mtDNA content was calculated relative to nDNA, mtDNA/nDNA = 2^(CTnDNA − CTmtDNA)^.

### Measurement of mitochondrial membrane potential (Δψ) and ATP concentration

TMRM (ThermoFisher Scientific) and MitoTracker Red CMXRos (ThermoFisher Scientific), which are lipophilic cationic fluorophores that accumulate in the mitochondria in proportion to Δψ (*41*, *69*), were used to monitor the Δψ. The cells were stained by addition of these dyes to the culture medium at 100 nM for 30 min at 37°C. Subsequently, the cells were stained with the Hoechst 33342 nuclear dye at 1:500 dilution in PBS, washed with PBS, and visualized using a Zeiss Axio Observer Z1 equipped with a digital camera (ORCA-ER C4742-80). The intensity of the fluorescence signal was quantified using ImageJ software or QuPath software.

To measure ATP concentration, the cells were seeded on Corning 96-well flat clear bottom white polystyrene tissue culture treated microplates (Corning Life Sciences, Tesksbury, MA) at 10,000 cells/cm^2^. In 2 to 3 days, the ATP concentration was measured using the Luminescent ATP Detection Assay Kit (Abcam, Cambridge, MA, catalog no. ab113849). Briefly, the cells were lysed using 28-μl lysis buffer for 10 min at room temperature (RT) on a shaking platform at 400 revolutions per minute. Subsequently, 3 μl of the lysate was transferred to another microplate for measuring the protein concentration using the Pierce Coomassie (Bradford) Protein Assay Kit according to the manufacturer’s protocol (ThermoFisher Scientific). Next, 25 μl of substrate was mixed with the remaining 25 μl of lysate and incubated for 15 min at RT in the dark. Luminescence absorbance was measured using the Synergy 4 microplate plate reader (BioTek, Winooski, VT), and the ATP concentration was calculated on the basis of the standard curve generated with ATP solution at 0 to 50 μM. The concentration of ATP was then reported as nanomole per milligram of protein by normalizing the ATP concentration to the protein concentration.

### Mitochondrial respiration and extracellular acidification

The OCR was measured using the XFe24 or XFe96 extracellular flux analyzer (Agilent, Santa Clara, CA). The cells were seeded at 14,000 cells per well in 24-well tissue culture plate or 8000 cells per well in 96-well tissue culture plate 24 hours before running the flux analyzer.

To assess mitochondrial respiration rate, the cells were incubated in XF base medium (Agilent) supplemented with 10 mM glucose, 1 mM pyruvate, and 2 mM glutamine for 1.5 hours. Subsequently, OCR was measured after addition of 1 μM oligomycin, 1.5 μM FCCP, and a mixture containing 0.5 μM each of antimycin A and rotenone. After the seahorse measurements were completed, total cellular content was measured using a CyQUANT Cell Proliferation Assay kit (ThermoFisher Scientific, catalog no. C7026), and the OCR values were normalized to (number of cells × mtDNA/nDNA).

### Single-cell gel electrophoresis assay (Comet assay)

To measure the extent of DNA damage, single-cell gel electrophoresis Comet Assay was performed according to the manufacturer’s instructions (Cell Bioloabs Inc., San Diego, CA). Briefly, agarose gel was heated in 95°C water bath for 10 min and then slowly cooled to 37°C, and 75 μl of agarose was spread on a OxiSelect glass slide and incubated at 4°C for 15 min to create a base layer. Subsequently, 75 μl of cells in agarose (10^5^ cells/ml) was spread on the base layer and incubated at 4°C for 15 min before submerging the slide in OxiSelect lysis buffer at 4°C for 45 min. After lysis, the slides were submerged in an alkaline solution [300 mM NaOH and 1 mM EDTA (pH > 13)] for 1 hour at 4°C before performing electrophoresis at 1 V/cm for 30 min in the alkaline solution. The slides were washed three times with deionized water (2 min each wash at 4°C) and once in 70% ethanol for 5 min and then allowed to dry overnight. The next day, slides were stained with OxiSelect Vista Green DNA dye for 15 min, and images were acquired using a Zeiss Axio Observer Z1 equipped with a digital camera (ORCA-ER C4742-80). The intensity of the Vista Green dye in the head and tail of the comet was measured using the ImageJ software with the OpenComet plugin (*70*), and the comet tail parameters were calculated as follows: tail DNA% = 100 × tail DNA intensity/total cell DNA intensity.

### Measurement of intracellular protein content

Cells (50 × 10^3^ to 100 × 10^3^) were centrifuged in 1.5-ml conical tubes at 300*g* for 5 min and then resuspended in 10 μl of radioimmunoprecipitation assay (RIPA) buffer (ThermoFisher Scientific). Lysate (1 μl) was diluted in 100 μl of PBS, and the protein concentration was measured using the Pierce Coomassie (Bradford) Protein Assay Kit according to the manufacturer’s protocol (ThermoFisher Scientific). Absorbance at 595 nm was measured using the Synergy 4 microplate plate reader (BioTek).

### Measurement of cell radius

To measure the cellular radius, the cells were detached from the surface using 0.25% Trypsin-EDTA and centrifuged at 300*g* for 5 min. The cell pellet was resuspended in PBS, and 10 μl was inserted in hemocytometer. Phase images were acquired using EVOS FL inverted digital microscope (ThermoFisher Scientific), and the cellular area was measured using the ImageJ software. Cell radius was calculated using the following equation: area = π × *R*^2^.

### RNA-seq and pathway analysis

RNA was isolated using the RNeasy Mini Kit (QIAGEN) according to the manufacturer’s protocol before library preparation. The quality of total RNA was assessed using the Agilent Fragment Analyzer, and concentration of each sample was measured using the High Sense RNA Qubit Fluorescence assay (Invitrogen, Carlsbad, CA). RNA libraries were prepared following the Illumina Stranded Total RNA library prep kit with a ribosomal removal step. Following library preparation, the concentration of each library was determined with a Qubit high sense DNA assay, and library quality was determined by the Agilent Fragment Analyzer. Final libraries were then pooled to 10 nM, and the concentration of the pool was determined using the Kapa Biosystems Universal qPCR kit (Roche, Wilmington, MA). After dilution to 300 pM and denaturing with NaOH, the pooled library was loaded onto a NovaSeq6000 SP flow cell (PE50; Illumina, San Diego, CA) for sequencing. Sequencing reads were preprocessed by FastQC (*71*) for sequencing quality control and mapped to the human GRCh38.p7 reference genome and the corresponding GENCODE v25 annotation database using TopHat2 (*72*). RSeQC (*73*) was applied to mapped BAM files for identifying potential RNA-seq library preparation problems. The read aligner, Subread (*74*), was used to quantify the number of reads for each gene. Differential gene expression analysis was performed using DESeq2 (*75*), a variance analysis package developed to infer the statistically significant difference in RNA-seq data, and biological hypothesis was tested using generalized linear model implemented in DESeq2 by constructing corresponding contrast, where multiple testing correction was applied.

Pathway analysis was performed with the GSEA method (3.0) (*76*). The preranked tool was chosen to run the analysis using a ranked gene list obtained from specific statistical comparisons ran on DESeq2. Pathway analysis was run against MSigDB, a collection of annotated and curated gene set repositories offered by the developer of GSEA (Broad Institute of MIT and Harvard). This particular run used C2 of version 6.1 collection, containing 1329 gene sets from various well-known and up-to-date pathway databases such as BioCarta, Kyoto Encyclopedia of Genes and Genomes (KEGG), and Reactome among others.

### Generation of LAKI-NANOG transgenic mice

The transgenic mice capable of NANOG expression upon Dox administration (C57BL/6; *Col1a1 ^tetO-Nanog/+^*, *ROSA26^rtTA/rtTA^*) (*24*) were provided by the laboratory of M. Serrano at The Barcelona Institute of Science and Technology, Barcelona, Spain. LAKI (C57BL/6, *Lmna^G609G/G609G^*) (*12*) fast aging mice were generated by Carlos López-Otín at the University of Oviedo, Spain and were donated by D. Lamming at the School of Medicine Madison, University of Wisconsin-Madison, Wisconsin. LAKI and NANOG transgenic mice were crossed by IVF at the Roswell Park Gene Targeting and Transgenic Shared Resource Facility to obtain the LAKIN genotype (*Col1a1 ^tetO-Nanog/+^*, *ROSA26^rtTA/rtTA^*, *Lmna^G609G/+^; Col1a1 ^tetO-Nanog/ tetO-Nanog^*, *ROSA26^rtTA/rtTA^*, *Lmna^G609G/+^; Col1a1 ^tetO-Nanog/+^*, *ROSA26^rtTA/+^*, *Lmna^G609G/+^; Col1a1 ^tetO-Nanog/ tetO-Nanog^*, ROSA26*^rtTA/+^*, *Lmna^G609G/+^*), which is the fast aging progeria mouse model that is capable of expressing NANOG upon Atridox administration. Two controls were used in the experiments: (i) the LAKI littermates (*Col1a1^+ /+^*, *ROSA26^rtTA/rtTA^*, *Lmna^G609G/+^; Col1a1^+/+^*, *ROSA26^+/rtTA^*, *Lmna^G609G/+^; Col1a1^+/+^*, *ROSA26^+/+^*, *Lmna^G609G/+^*) injected with Atridox (10 males and 2 females) or (ii) the LAKIN genotypes injected with the Atrigel vehicle without Dox (4 males and 3 females). The experiments were performed with both male and female mice at the age of 10 months, when the heterozygous progeria mice (*Lmna^G609G/+^*) show signs of premature aging, such as kyphosis, muscle loss, and grey hair (*77*).

For the *Lmna^+/+^* controls at the age of 10 months, we used mice with NANOG genotype (*Col1a1 ^tetO-Nanog/+^*, *ROSA26^rtTA/rtTA^*, *Lmna^+/+^; Col1a1 ^tetO-Nanog/ tetO-Nanog^*, *ROSA26^rtTA/rtTA^*, *Lmna^+/+^; Col1a1 ^tetO-Nanog/+^*, *ROSA26^rtTA/+^*, *Lmna^+/+^; Col1a1 ^tetO-Nanog/ tetO-Nanog^*, *ROSA26^rtTA/+^*, *Lmna^+/+^*) that were administered with Dox (denoted as WN). The control WT animals were either (*Col1a1 ^+/ +^*, *ROSA26^rtTA/rtTA^*, *Lmna^+/+^; Col1a1^+ /+^*, *ROSA26^rtTA/+^*, *Lmna^+/+^; Col1a1^+/ +^*, *ROSA26^+/+^*, *Lmna^+/+^*) administered with Atridox (4 males and 3 females) or the NANOG genotype administered with the Atrigel vehicle without Dox (4 males and 5 females).

In vitro fertilization (IVF) using the method from Center for Animal Resources and Development (CARD) was done as described previously (*78*). Briefly, frozen sperm from a male heterozygous for the Nanog, heterozygous for the rtTA, and homozygous for the Lnma mutation (*Col1a1 ^tetO-Nanog/+^*, *ROSA26^rtTA/+^*, *Lmna^G609G/G609G^*) was used for multiple rounds of IVF with females of various genetic backgrounds to produce the maximum amount of *Lmna^G609G/+^* progeny possible because breeding by traditional means did not yield enough progeny to power our experiments. Specifically, 8- to 12-week-old females were super-ovulated with 7.5 IU of pregnant mares’ serum (ProSpec, Brunswick, NJ) followed by 7.5 IU of human chorionic gonadotropin (HCG; ProSpec) given 46 to 48 hours later. Eggs were harvested 15 to 17 hours after HCG, and clutches of eggs were put directly into the CARD fertilization medium drops (Cosmo Bio, Carlsbad, CA) and incubated for 30 to 60 min at 37°C. Frozen sperm was thawed and plunged into a drop of CosmoBio FERTIUP medium (Cosmo Bio) for 30 min. After these incubations, 10 μl of sperm from the FERTIUP drop was pipetted into the fertilization drop with the eggs. After 3 hours, embryos were washed several times with Cook medium (Cook Medical, Bloomington, IN) and incubated overnight in Cook medium to assess fertilization rate before surgically transferring two-cell embryos the following day.

The mice were housed in a temperature-controlled animal holding room (20.5° to 23.9°C) with 12-hour light/12-hour dark cycle between 6:00 and 18:00 standard time. Food and water were provided ad libitum, and the animals were excluded from the experiments if a weight loss of >20% occurred. All animal experiments were approved by the Institutional Animal Care and Use Committee (IACUC) at the University at Buffalo.

### Genotyping

To perform genotyping, tail snips or ear punches were acquired from 2- to 3-week-old animals according to the standard IACUC protocols. To extract DNA, the tails were digested in 75 μl of alkaline lysis solution (25 mM NaOH and 0.2 mM EDTA) for 45 min at 95°C. The alkaline lysis solution was neutralized by 75 μl of neutralization buffer (40 mM tris-HCl). DNA amplification was performed by PCR or Sanger sequencing using the following primers: Nanog primers: CCCTCCATGTGTGACCAAGG (common), GCACAGCATTGCGGACATGC (wt), and GCAGAAGCGCGGCCGTCTGG (tg); rtTA primers: AAAGTCGCTCTGAGTTGTTAT (common), GCGAAGAGTTTGTCCTCAACC (mut F), and GGAGCGGGAGAAATGGATATG (mut R); and LMNA primers: GGTTCCCACTGCAGCGGCTC (mm-lmna forward exon 1) and GGACCCCACTCCCTTGGGCT [mm-lmna reverse (intron)].

Each PCR reaction required 17.5 μl of H_2_O, 3.125 μl of 10x Buffer (QIAGEN), 1.625 μl of 25 mM MgCl_2_, 0.5 μl of 25 mM dNTPs, 0.5 μl of HotStarTaq DNA polymerase (5 U/μl; QIAGEN), 0.625 μl of primer, and 3 μl of DNA. The PCR reaction was run using Veriti thermocycler (Applied Biosystems, Foster City, CA), and the PCR products were subjected to electrophoresis in 2% agarose gel at 210 V for 90 min. The expected band sizes for NANOG are 331–base pair (bp) WT and 551-bp transgenic, and for rtTA are 500-bp WT and 250-bp transgenic. The LMNA PCR product at 300-bp band size was purified using the QIAquick PCR purification kit (QIAGEN) and was sequenced by Sanger sequencing at the Roswell Park Genomic Shared Resources.

### Intramuscular induction of NANOG

NANOG expression was induced by intramuscular injection of Atridox (DenMat, Lompoc, CA) to the TA muscle, which allowed slow release of Dox in the TA muscle. Atridox is a mixture of Atrigel delivery system and Dox hyclate. Specifically, the Atrigel delivery system containing 450 mg 36.7% (w/w) poly(d,l-lactide) (PLA) was dissolved in 63.3% (w/w) *N*-methyl-2-pyrrolidone (NMP) solvent and then mixed with 50 mg of Dox hyclate (equivalent to 42.5 mg of Dox) to make Atridox. Before injection, the animals were anesthetized using isoflurane, and the skin on top of the TA muscle was shaved and disinfected with 70% ethanol. Then, 25 μl of Atridox was intramuscularly injected at 30° to 45° angle to the TA muscle using 31-gauge needle. After injection, the liquid PLA/NMP solidified and released Dox gradually over a period of 2 to 3 weeks (*46*, *47*). For control, 25 μl of Atrigel without Dox was injected to the TA muscle. The animals were sacrificed and the TA muscle was isolated at 3 or 5 weeks after the atridox injection to quantify the abundance of myogenic progenitors and the % SA-β-Gal–positive myogenic progenitors in Fig. 9 (C to E). No significant difference was observed between the two time points, and therefore, the two time points were pooled together (WT, 12 mice at 3 weeks and 4 mice at 5 weeks; WTN, 6 mice at 3 weeks and 6 mice at 5 weeks; LAKI, 12 mice at 3 weeks and 7 mice at 5 weeks; LAKIN, 11 mice at 3 weeks and 5 mice at 5 weeks). The quantifications of eMyHC^+^ myofibers in Fig. 9 (G and H) also did not show a difference comparing the two different time points, and therefore, the time points were pooled together (WT, 6 mice at 3 weeks and 2 mice at 5 weeks; WTN, 4 mice at 3 weeks and 3 mice at 5 weeks; LAKI, 10 mice at 3 weeks and 4 mice at 5 weeks; LAKIN, 6 mice at 3 weeks and 3 mice at 5 weeks).

### Cryopreservation

Each muscle was isolated within 10 min after euthanizing the mouse using CO_2_ chamber, and the tissue was fixed in 10% formalin (Sigma-Aldrich) for 6 hours. Subsequently, the tissues were washed with PBS and transferred to PBS and 0.1 M Glycine (VWR) for 1 hour before dehydrating them in two steps in PBS containing 15 and 30% Sucrose (Ward’s Science, Rochester, NY). The tissues were then immersed in tissue embedding medium (OCT, Sakura Finetek, Torrance, CA) and frozen in 2-methylbutane (Sigma-Aldrich) chilled with dry ice. The frozen blocks were stored at −80°C until they were transferred to a −20°C cryostat to obtain 10-μm-thick tissue sections that were placed on positively charged glass slides (Stellar Scientific, Baltimore, MD). After cutting, tissue sections were allowed to dry at RT for 5 min and stored in −80°C.

### Immunostaining

The tissue sections from −80°C were washed three times in PBS to remove OCT before permeabilization in −20°C methanol (Sigma-Aldrich) for 10 min. Antigen retrieval was performed by immersing the slides in R-Buffer A (Electron Microscopy Sciences, Hatfield, PA) while raising the temperature to 95°C for 20 min and allowing them to cool down gradually over a period of 2 hours. Peroxidases were then blocked using tyramide H_2_O_2_ solution (Alexa Fluor 555 Tyramide SuperBoost Kit, ThermoFisher Scientific) for 30 min before blocking with PBS with 5% (w/v) goat serum and 5% (w/v) BSA for 1 hour. The samples were then blocked with Tyramide Blocking Buffer for 1 hour and mouse immunoglobulin G blocking reagent [Mouse on Mouse (MOM), Vector Labs, Burlingame, CA] for 1 hour according to the manufacturer’s protocol. The tissues were then incubated overnight at 4°C with primary antibodies diluted in MOM diluent: Pax7 (1:10 dilution; Developmental Studies Hybridoma Bank, Iowa City, IA, catalog no. AB_528428), eMyHC (1:100 dilution; Santa Cruz Biotechnology, Dallas, Texas, catalog no. sc-53091 Alexa Fluor 647-conjugated), laminin (1:200 dilution; Sigma-Aldrich, catalog no. L9393), Desmin (1:200 dilution; Cell Signaling Technology, Danvers, MA, catalog no. 5332), phospho-histone H2A.X (Ser^139^) (1:200 dilution; Cell Signaling Technology, catalog no. 9718), Nanog (1:200 dilution; Cell Signaling Technology, catalog no. 3580, for human cells and Abcam, catalog no. 80892, for mouse cells), Ki67 (1:200 dilution; Abcam, catalog no. ab15580), H3K9me3 (1:200 dilution; Abcam, catalog no. ab8898), H3K27me3 (1:200 dilution; Cell Signaling Technology, 9733), H4K20me2 (1:200 dilution; Abcam, catalog no. ab9052), and lamin A/C (1:100 dilution; Cell Signaling Technology, catalog no. 4777). The next day, the samples were washed with PBS and stained with goat anti-mouse secondary antibody from the Alexa Fluor 555 Tyramide SuperBoost Kit (ThermoFisher Scientific, catalog no. B40913) according to the kit protocol. Subsequently, the samples were stained with Alexa Fluor 488 or 647–conjugated goat anti-rabbit secondary antibodies for 1 hour.

For cells, they were fixed for 10 min in 4% paraformaldehyde before permeabilization for 10 min with 0.1% (v/v) Triton X-100/PBS and blocking for 1 hour with blocking buffer [5% (v/v) goat serum in 0.01% (w/v) Triton X-100/PBS] at RT. The samples were incubated overnight at 4°C with the primary antibodies diluted in blocking buffer at the dilutions indicated above. Subsequently, the cells were stained for 1 hour at RT with Alexa Fluor 568 or 488–conjugated goat anti-rabbit or goat anti-mouse secondary antibodies, and nuclei were counterstained with Hoechst 33342 nuclear dye for 5 min at RT. To visualize actin filaments, the cells were stained with Alexa Fluor 647 Phalloidin (1:50 dilution; ThermoFisher Scientific, catalog no. A22287) for 4 hours at RT.

Images were acquired using a Zeiss Axio Observer Z1 (LSM 510) equipped with a digital camera (ORCA-ER C4742-80). ImageJ software and QuPath software were used to measure the area and fluorescence intensity. The extent of nuclear deformity was quantified by calculating nuclear circularity as 4π × area × perimeter^−2^,which is equal to 1 for a perfectly round object.

For each animal, fluorescence intensity was measured in at least three independent sections, each containing 1000 to 3000 cells, and reported as the % positive cells/myofibers (one bubble represents one sample/animal in the scatter dot plot). For cells in culture, the fluorescence intensity was measured in at least three independent samples each containing 200 to 500 cells.

### Senescence-associated β-galactosidase

The SA-β-Gal staining solution was prepared according to the manufacturer’s protocol (ThermoFisher Scientific, catalog no. K146501). The fixed cells or tissues cross sections were incubated with the SA-β-Gal staining solution overnight at 37°C. On the next day, the samples were washed with PBS and imaged using Zeiss Axio Observer Z1 (LSM 510) equipped with an Axiocam MRC camera. SA-β-Gal intensity was calculated as the SA-β-Gal–positive area divided by the total cell area.

### Western blotting

RIPA lysis buffer (ThermoFisher Scientific) containing Halt Protease Inhibitor Cocktail (ThermoFisher Scientific) and Halt Phosphatase Inhibitor Cocktail (ThermoFisher Scientific) was used for lysing cells. The lysed cells were centrifuged at 14,000*g* for 10 min to pellet the lipids, and the supernatants were then transferred to new tubes. After addition of 42 mM dithiothreitol and Blue Loading Buffer (Cell Signaling Technology) to the samples, they were denatured at 95°C for 5 min, and then cellular proteins were separated on the basis of the molecular weight by electrophoresis through 8 to 12% polyacrylamide gel. After transferring proteins to nitrocellulose membranes (Trans-Blot Turbo Transfer System; Bio-Rad, Hercules, CA), the membranes were blocked by 5% nonfat dry milk in Tris-buffered saline with 0.1% Tween® 20 detergent (TBST) buffer (20 mM tris, 150 mM NaCl, and 0.1% Tween 20) for 1 hour before probing for specific proteins at 4°C overnight with the following antibodies were diluted at 1:1000 ratio in TBST containing 1% BSA: NANOG (Cell Signaling Technology, catalog no. 3580, for human cells and Abcam, catalog no. 80892, for mouse cells), H3K9me3 (Abcam, catalog no. ab8898), H3K27me3 (Cell Signaling Technology, 9733), H4K20me2 (Abcam, catalog no. ab9052), H4 (Abcam, catalog no. ab10158), H3 (Cell Signaling Technology, catalog no. 4499), AMPKα (Cell Signaling Technology, catalog no. 2532), pAMPKα (T172, Cell Signaling Technology, catalog no. 2535), mTOR (Cell Signaling Technology, catalog no. 2983), p-mTOR (Ser^2448^; Cell Signaling Technology, catalog no. 5536), Parkin (Cell Signaling Technology, catalog no. 2132), PINK1 (Cell Signaling Technology, catalog no. 6946), Beclin-1 (Cell Signaling Technology, catalog no. 3495), LC3 (Cell Signaling Technology, catalog no. 12741), ATG12 (Cell Signaling Technology, catalog no. 4180), ATG7 (Cell Signaling Technology, catalog no. 8558), ATG5 (Cell Signaling Technology, catalog no. 12994), phospho-ATM (Ser1981, Cell Signaling Technology, catalog no. 5883), ATM (Cell Signaling Technology, catalog no. 2873), DNA-dependent protein kinase (DNA-PK) (Cell Signaling Technology, catalog no. 4602), Ku80 (Cell Signaling Technology, catalog no. 2180), Mre11 (Cell Signaling Technology, catalog no. 4847), Rad50 (Cell Signaling Technology, catalog no. 3427), X-Ray Repair Cross Complementing 4-like factor (XLF) (Cell Signaling Technology, catalog no. 2854), H2A (Cell Signaling Technology, catalog no. 12349), and phospho-histone H2A.X (Ser^139^; Cell Signaling Technology, catalog no. 9718), as well as loading controls glyceraldehyde-3-phosphate dehydrogenase (Cell Signaling Technology, catalog no. 2118) and β-tubulin (Abcam, catalog no. ab6046). The protein bands were detected using horseradish peroxidase–conjugated secondary antibodies (Cell Signaling Technology) and SuperSignal West Pico PLUS chemiluminescence substrate (ThermoFisher Scientific). The protein bands were visualized using the ChemiDoc MP imaging system (Bio-Rad), and the images were analyzed using the Image Lab software (Bio-Rad).

### Statistical analysis

Statistical significance for each experiment was assessed by performing one-way or two-way analysis of variance (ANOVA) analysis followed by Tukey’s multiple comparisons test using GraphPad Prism version 8 software, and the values were considered statistically significant if *P* < 0.05. Here, $ denotes *P* < 0.05 as compared to all other samples, # denotes *P* < 0.05 as compared to senescent cells, and * denotes *P* < 0.05 in a paired comparison. The data were shown as means ± 95% confidence interval (CI) of one representative experiment, where each bubble represents one cell; or means ± SD of multiple independent experiments, where each bubble represents one donor. The cells from different donors were not mixed together; instead, we performed experiments with cells for each donor separately. Each experiment was repeated at least three times with cells from three independent donors to ensure reproducibility of the results.

## Notes

View/request a protocol for this paper from *Bio-protocol*.
