# Supplementary material for: Ameliorating the hallmarks of cellular senescence in skeletal muscle myogenic progenitors in vitro and in vivo
Source: Sci Adv. 2021 Sep 3;7(36):eabe5671. doi: 10.1126/sciadv.abe5671 (PMC8442867; doi:10.1126/sciadv.abe5671)
Supplement: Supplementary file 1 — Figs. S1 to S6 [file sciadv.abe5671_sm.pdf]

Science Advances
AAAS

Supplementary Materials for

Ameliorating the hallmarks of cellular senescence in skeletal muscle myogenic progenitors in vitro and in vivo

Aref Shahini, Nika Rajabian, Debanik Choudhury, Shahryar Shahini, Kalyan Vydiam, Thy Nguyen, Joseph Kulczyk, Tyler Santarelli, Izuagie Ikhapoh, Yali Zhang, Jianmin Wang, Song Liu, Aimee Stablewski, Ramkumar Thiyagarajan, Kenneth Seldeen, Bruce R. Troen, Jennifer Peirick, Pedro Lei, Stelios T. Andreadis*

*Corresponding author. Email: sandread@buffalo.edu

Published 3 September 2021, *Sci. Adv.* **7**, eabe5671 (2021)
DOI: 10.1126/sciadv.abe5671

The PDF file includes:

Figs. S1 to S6

Other Supplementary Material for this manuscript includes the following:

Data files S1 to S8

Supplementary Figure 1

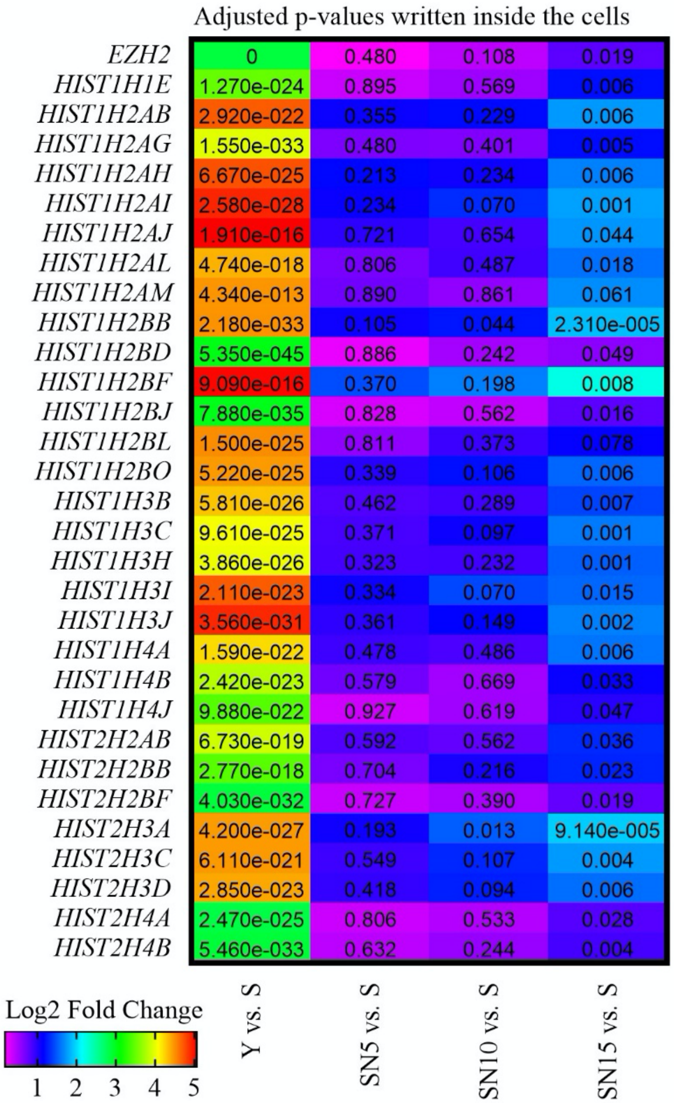

Supplementary Figure 1: Heat map of mRNA expression level of histone proteins and EZH2 histone methyl transferase. The color represents the Log2 fold increase and the number in each box represents the adjusted p-value.

Supplementary Figure 2

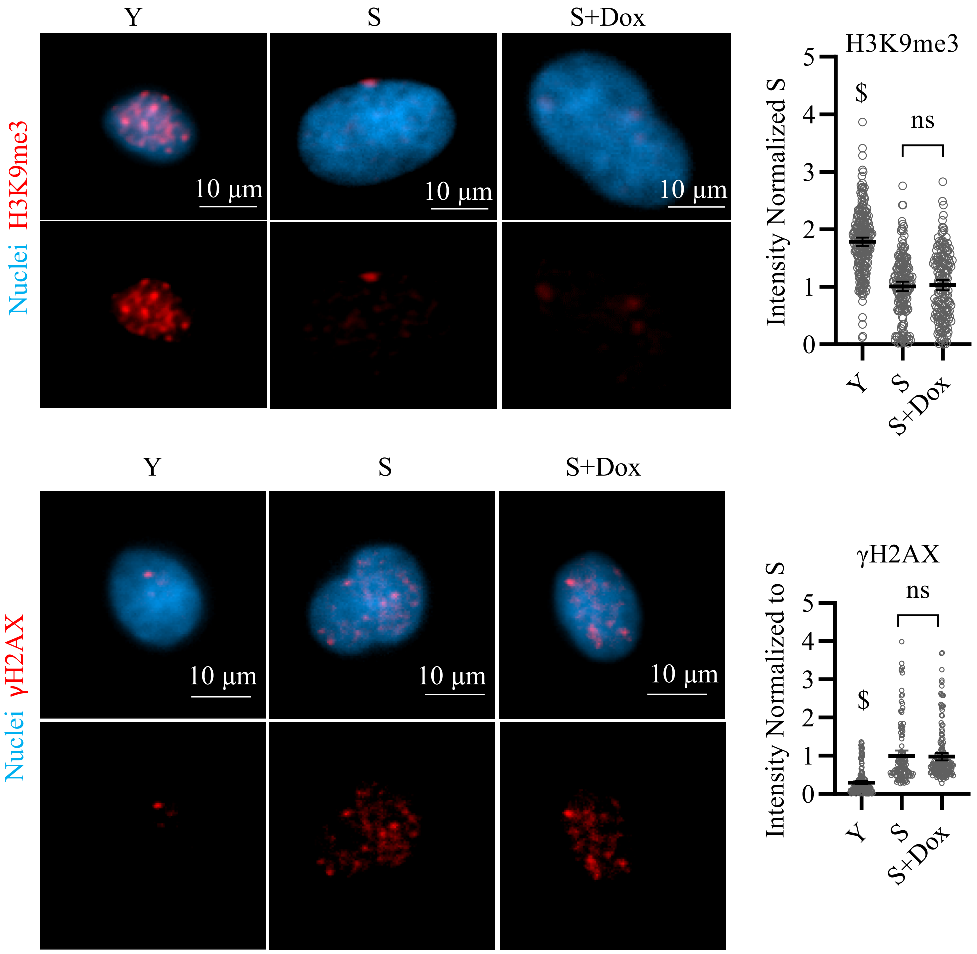

Supplementary Figure 2: Dox had no effect on heterochromatin marks and DNA damage response. Control S cells (without NANOG transgene) were treated with Dox at 1µg/ml concentration for 15 days. A) Dox treatment did not change the levels of heterochromatin mark (H3K9me3) in control senescent cells. B) Dox treatment did not change the levels of DNA damage marker (γH2AX) in control senescent cells. $ signs denote $P<0.05$ as compared to all other samples.

Supplementary Figure 3

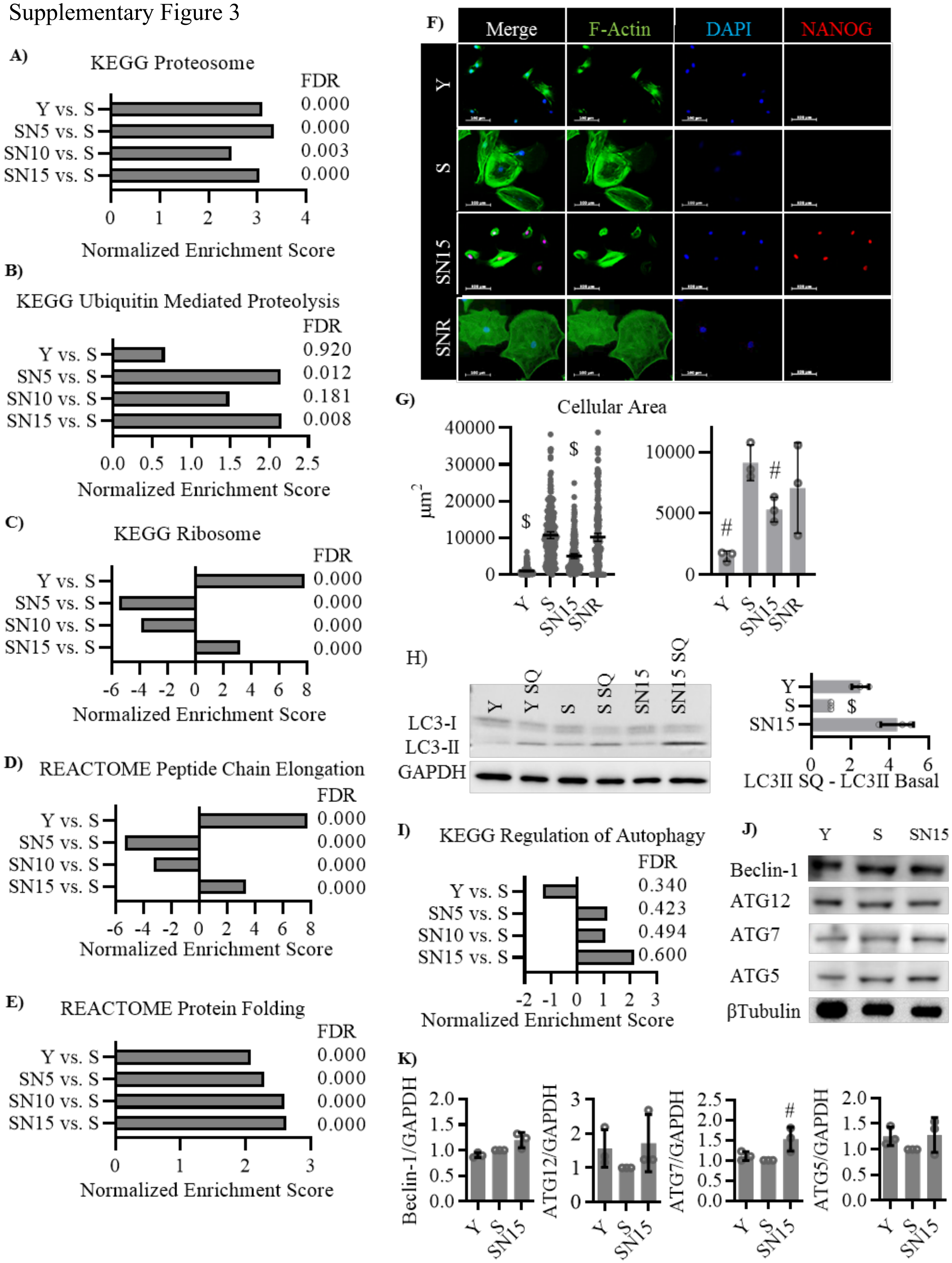

Supplementary Figure 3: Restoration of proteostasis by NANOG in senescent cells. (A-E) GSEA analysis suggests that **(A, B)** NANOG expression for 5-15 days in senescent cells leads to significant enrichment of proteasome compartments as well as ubiquitin mediated proteolysis pathway by NANOG; **(C, D)** NANOG initially downregulates ribosome and peptide elongation pathway but eventually increases the activity of these pathways in senescent cells; **(E)** NANOG expression restores protein folding processes in senescent cells. **(F)** Immunostaining of Y, S, SN15, and SNR myoblasts for NANOG (red), nuclei (blue) and F-Actin (green). **(G)** Quantification of the cellular cytoplasmic area; data shown as mean ± 95% CI for >200 cells per condition and mean ± STD for three donors. **(H)** Western blotting analysis of LC3 protein upon starvation+chloroquine (SQ) treatment for 1.5hr and quantification of the increase in LC3-II after SQ treatment as a metric of autophagosome formation. **(I)** GSEA analysis suggests no statistically significant difference in the transcriptional regulation of autophagy pathway. **(J-K)** Western blotting quantification of autophagy proteins. $ denotes $p<0.05$ as compared to all other samples. # denotes $p<0.05$ as compared to S.

Supplementary Figure 4

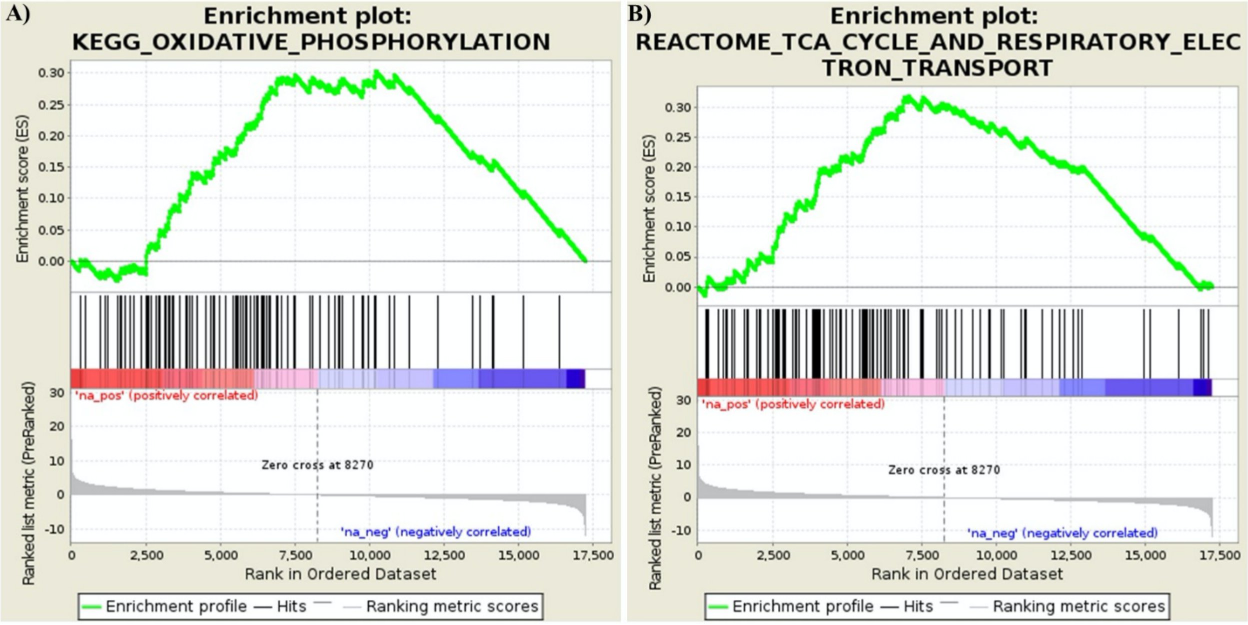

Supplementary Figure 4: (A&B) Enrichment plots for Oxidative Phosphorylation pathway as well as TCA Cycle and Respiratory Electron Transport pathway comparing SN15 vs S.

Supplementary Figure 5

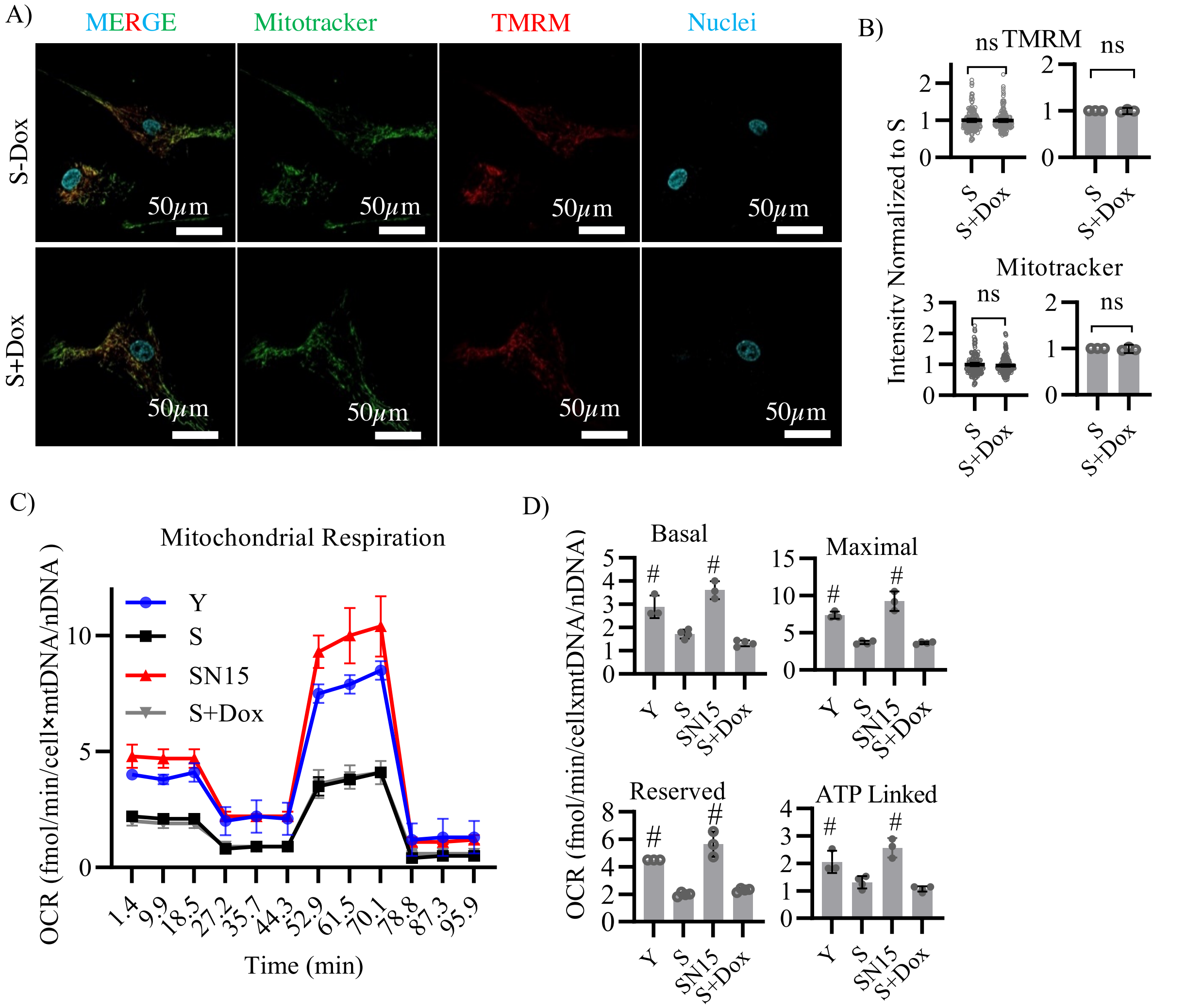

Supplementary Figure 5: Dox had no effect on mitochondrial function. Control senescent cells (without NANOG transgene) were treated with Dox at the concentration of 1μg/ml for 15 days (S+Dox). **A&B)** Assessing mitochondrial membrane potential by staining for Mitotracker (green) and TMRM (red) dyes and quantifying the fluorescence intensity. **C&D)** Measurement of Oxygen Consumption Rate (OCR) in a Mitostress test to assess the mitochondrial respiration capacity. # signs denote $P<0.05$ as compared to S.

Supplementary Figure 6

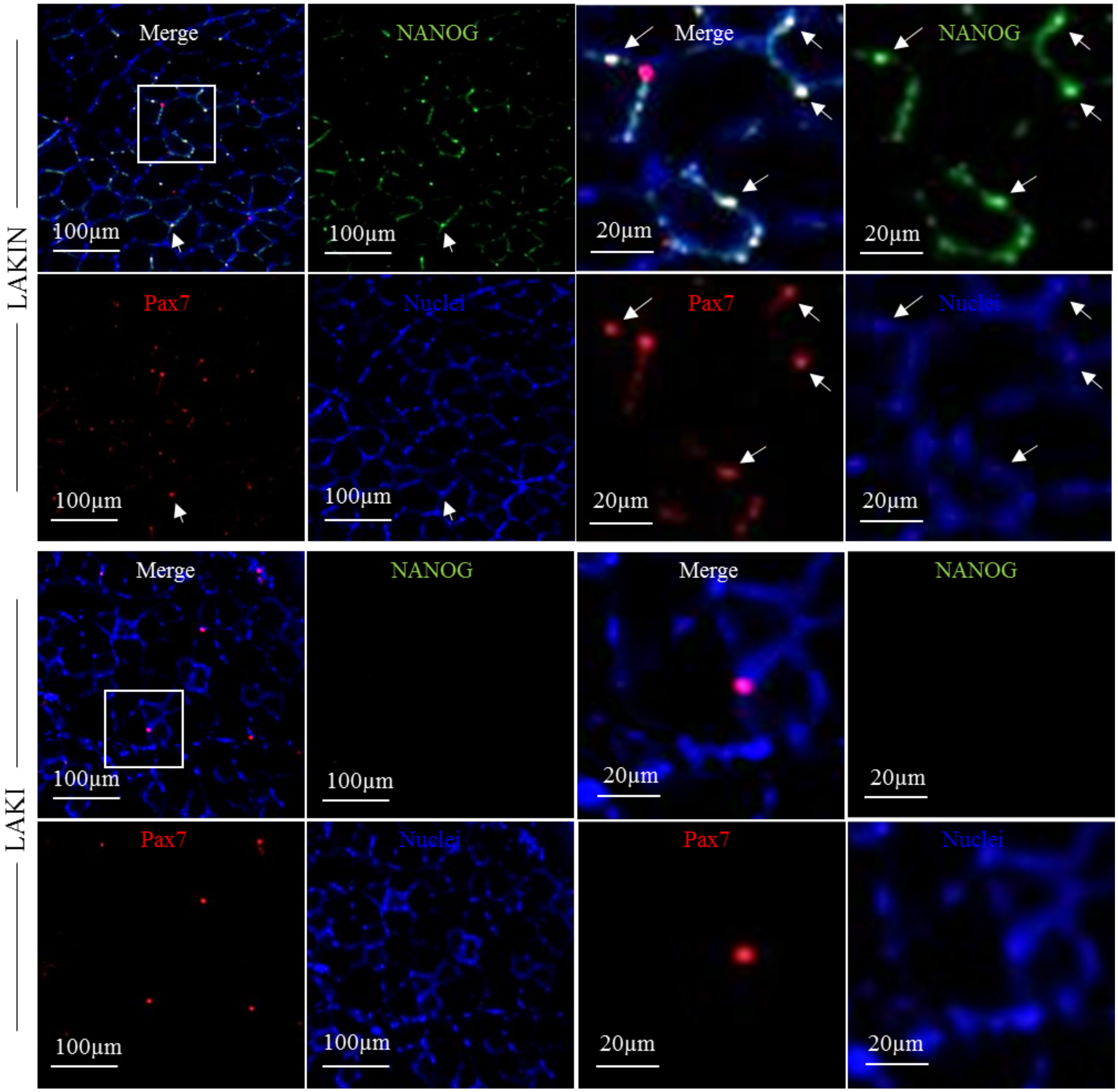

Supplementary Figure 6: Immunostaining of LAKI and LAKIN muscle cross-sections for NANOG (green) and Pax7 (red) near the Atridox injection site. The arrows point to the Pax7+ Nanog+ cells. The white square is magnified on the right.
